# Interaction of 4′-methylflavonoids with biological membranes, liposomes, and human albumin

**DOI:** 10.1038/s41598-021-95430-8

**Published:** 2021-08-06

**Authors:** Aleksandra Włoch, Paulina Strugała-Danak, Hanna Pruchnik, Agnieszka Krawczyk-Łebek, Karolina Szczecka, Tomasz Janeczko, Edyta Kostrzewa-Susłow

**Affiliations:** 1 Department of Physics and Biophysics, Wrocław University of Environmental and Life Sciences, C. K. Norwida 25, 50-375 Wrocław, Poland; 2 Department of Chemistry, Wrocław University of Environmental and Life Sciences, C. K. Norwida 25, 50-375 Wrocław, Poland

**Keywords:** Biophysics, Chemical biology

## Abstract

The aim of the study was to compare the impact of three synthesized chemical compounds from a group of methylated flavonoids, i.e. 2′-hydroxy-4-methylchalcone (**3**), 4′-methylflavanone (**4**), and 4′-methylflavone (**5**), on a red blood cell membranes (RBCMs), phosphatidylcholine model membranes (PC), and human serum albumin (HSA) in order to investigate their structure–activity relationships. In the first stage of the study, it was proved that all of the compounds tested do not cause hemolysis of red blood cells and, therefore, do not have a toxic effect. In biophysical studies, it was shown that flavonoids have an impact on the hydrophilic and hydrophobic regions of membranes (both RBCMs and PC) causing an increase in packing order of lipid heads and a decrease in fluidity, respectively. Whereas, on the one hand, the magnitude of these changes depends on the type of the compound tested, on the other hand, it also depends on the type of membrane. 4′-Methylflavanone and 4′-methylflavone are located mainly in the hydrophilic part of lipid membranes, while 2′-hydroxy-4-methylchalcone has a greater impact on the hydrophobic area. A fluorescence quenching study proved that compounds (**3**), (**4**) and (**5**) bind with HSA in a process of static quenching. The binding process is spontaneous whereas hydrogen bonding interactions and van der Waals forces play a major role in the interaction between the compounds and HSA.

## Introduction

At present, at the center of numerous scientific studies are natural compounds of plant origin which are called phytochemicals. One commonly found class of these compounds, which is widely distributed in the world of plants, are flavonoids, namely a group of compounds naturally present mainly in fruit and vegetables, as well as in tea, cocoa and wine. They exhibit a wide spectrum of biological activities, including antioxidant, anti-inflammatory, anti-radical, anti-cancer, antimicrobial, antimalarial^1–6^. Their biological activity is related to their molecular structure and depends on both the number and place of attachment of functional groups (e.g. hydroxyl groups) in the molecule, as well as the possibility of interacting with a biological membrane which is the first natural barrier in reaching the interior of cells by these components^7,8^. In nature, flavonoids often occur in plants in either glycosylated or methylated form, because these structures are more stable, bioavailable, and also bioactive. Numerous scientific studies have shown that methylated flavonoids exhibit much better biological properties than their unmethylated forms^9–11^. For example, 7-hydroxy-8-methoxyflavone, unlike 7,8-dihydroxyflavone, did not affect the viability of endothelial cells and, therefore, had a cytoprotective effect, showing a strong antioxidant activity even in long-term therapies^12^. Flavonoids are supplied to the body in food. Their pro-health effect on a human body is closely related to their bioavailability, which consists of a number of factors, including the size of particles, their solubility, and the time of passing through the digestive tract. One of the factors connected with bioavailability is their transport based on the main human plasma protein, i.e. albumin (HSA). This protein serves many important functions in a human body. One of them stems from the fact that albumin maintains a high rate of penetration into interstitial fluids, which allows it to come into contact with most of the body's cells, making it an ideal carrier of various substances. Moreover, albumin has a natural affinity for many exogenous and endogenous substances, which improves the therapeutic activity of drugs associated. Determining the strength of binding between a potential drug and albumin is the basic factor that determines its health-promoting properties. The research conducted by Strugała et al. showed that polyphenolic extracts bind to human albumin, possibly by forming complexes via hydrogen and van der Waals bonds^3^. The results of these studies constitute the foundations of medical chemistry as far as pharmacokinetics and distribution of the tested compounds are considered.

As a result of the research focused on methylation of flavonoids, it was proved that this process leads to derivatives which are insusceptible to glucuronic acid or sulfate conjugation, thus protects methylated flavonoids from extensive, rapid hepatic metabolism^12–14^. In addition, methylation of a flavonoid aglycone significantly increases its metabolic stability and enhances the membrane transport, resulting in easier absorption, and, all in all, in greater oral bioavailability. For example, in vivo studies conducted by Walle et al. showed that 7-hydroxyflavone, 7,4′-dihydroxyflavone, and 5,7-dihydroxyflavone were not detected in the tissues, after having been administered to rats, while their methylated derivatives reached high level of concentration^15^. Despite numerous studies on the biological activity of flavonoids, the mechanism of their action at the cellular level is not fully explained. Only limited scientific studies have been conducted and their results show that flavonoids have an impact on the properties of membranes. However, there are no papers containing comprehensive structural studies explaining the influence of this group of compounds on the physical and chemical properties of a biological membrane. This above–mentioned issue would be of great importance due to the large potential of these compounds to be used in the treatment of many diseases.

The main goal of this study was to verify whether the substitution of 4**′**-methyl moiety of flavonoids belonging to three subgroups (chalcone, flavanone and flavone) has an impact on their localization and interactions with both biological membrane and biomolecules. For this purpose, the range of concentrations in which the tested compounds do not cause cytotoxic effect on erythrocyte cells was checked first. In order to determine the location of the interaction of flavonoids with a model lipid membrane (liposomes, PC) and a biological protein-lipid membrane (RBCMs), comprehensive biophysical studies (using fluorimetric and infrared spectroscopy methods) were carried out. Finally, it was checked whether the newly obtained flavonoids bind to the human serum albumin and the binding mechanism was meticulously analyzed.

## Results and discussion

### Synthesis of 2′-hydroxy-4-methylchalcone (3), 4′-methylflavanone (4), and 4′-methylflavone (5)

**Scheme 1:**
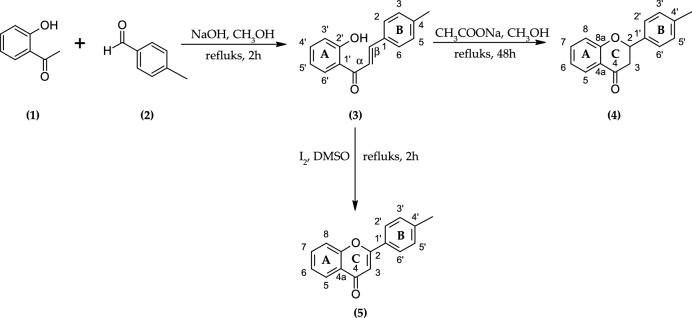
Synthesis of 2*′*-hydroxy-4-methylchalcone (**3**), 4*′*-methylflavanone (**4**), and 4*′*-methylflavone (**5**).

The compounds for experiments were received by chemical synthesis (Scheme 1). The first step was Claisen-Schmidt condensation between 2*′*-hydroxyacetophenone (**1**) and 4-methylbenzaldehyde (**2**). The chemical reagents were dissolved in methanol, and the reaction was carried out under alkaline conditions at high temperature^16–18^. 2*′*-Hydroxy-4-methylchalcone (**3**) was obtained with 65.5% yield. 4*′*-Methylflavanone (**4**) was received by cyclization of 2*′*-hydroxy-4-methylchalcone (**3**) in the presence of sodium acetate with 37.7% yield. 4*′*-Methylflavone (**5**) was obtained from of 2*′*-hydroxy-4-methylchalcone (**3**) by reaction with iodine excess with 70.0% yield^16^. The data describing obtained synthesis products are shown below and in the Supplementary Materials.

#### 2′-Hydroxy-4-methylchalcone (3)

C_16_H_14_O_2_, t_R_ 18.56; ^**1**^**H NMR** (acetone-d_6_) δ (ppm): 12.94 (1H, s, -OH), 8.27 (1H, d, *J* = 8.2 Hz, H-6*′*), 8.02 (1H, d, *J* = 15.4 Hz, H-β), 7.93 (1H, d, *J* = 15.4 Hz, H-α), 7.79 (2H, d, *J* = 8.0 Hz, H-2, H-6), 7.57 (1H, m, H-4*′*), 7.31 (2H, d, *J* = 7.9 Hz, H-3, H-5), 6,99 (2H, t, *J* = 8.4 Hz, H-3*′*, H-5*′*), 2.39 (3H, s, –CH_3_); ^**13**^**C NMR** (acetone-d_6_) δ (ppm): 195.05 (C=O), 164.53 (C-2*′*), 146.36 (C-β), 142.44 (C-1), 137.35 (C-4*′*), 133.03 (C-4), 131.34 (C-6*′*), 130.58 (C-3, C-5), 130.00 (C-2, C-6), 120.92 (C-1*′*), 120.35 (C-α), 119.79 (C-5*′*), 118.94 (C-3*′*), 21.51 (-CH_3_).

#### 4′-Methylflavanone (4)

C_16_H_14_O_2_, *t*_R_ 17.33;${[\alpha ]}_{ D}^{20}$ = 0 (c = 0,34 acetone); ^**1**^**H NMR** (acetone-d_6_) δ (ppm): 7.84 (1H, dd, *J* = 7.8, 1.2 Hz, H-5), 7.57 (1H, m, H-7), 7.47 (2H, d, *J* = 8.0 Hz, H-2*′*, H-6*′*), 7.26 (2H, d, *J* = 7.9 Hz, H-3*′*, H-5*′*), 7.08 (2H, dd, *J* = 14.0, 7.8 Hz, H-6, H-8), 5.59 (1H, dd, *J* = 13.0, 2.8 Hz, H-2), 3.15 (1H, dd, *J* = 16.7, 13.0 Hz, H-3_ax_), 2.83 (1H, dd, *J* = 16.7, 2.6 Hz, H-3_ eq_), 2.36 (3H, s, –CH_3_); ^**13**^**C NMR** (acetone-d_6_) δ (ppm): 191.93 (C-4), 162.49 (C-8a), 139.07, (C-1*′*), 137.39 (C-4*′*), 136.80 (C-7), 130.06 (C-3*′*, C-5*′*), 127.37 (C-5), 127.34 (C-2*′*, C-6*′*), 122.19 (C-6), 121.97 (C-4a), 118.93 (C-8), 80.29 (C-2), 44.94 (C-3), 21.17 (4*′*-CH_3_).

#### 4′-Methylflavone (5)

C_16_H_12_O_2_ , *t*_R_ 16.82; ^**1**^**H NMR** (acetone-d_6_) δ (ppm): 8.12 (1H, dd, *J* = 7.9, 1.6 Hz, H-5), 7.99 (2H, d, *J* = 8.3 Hz, H-2*′*, H-6*′*), 7.81 (1H, ddd, *J* = 8.7, 7.1, 1.7 Hz, H-7), 7.73 (1H, d, *J* = 8.4 Hz, H-8), 7.48 (1H, m, H-6), 7.41 (2H, d, *J* = 8.0 Hz, H-3*′*, H-5*′*), 6.82 (1H, s, H-3), 2.43 (3H, s, –CH_3_); ^**13**^**C NMR** (acetone-d_6_) δ (ppm): 177.87 (C-4), 164.10 (C-2), 157.15 (C-8a), 143.09, (C-4*′*), 134.73 (C-7), 130.63 (C-3*′*, C-5*′*), 129.99 (C-1*′*), 127.18 (C-2*′*, C-6*′*), 126.07 (C-6), 125.98 (C-5), 124.91 (C-4a), 119.21 (C-8), 107.37 (C-3), 21.44 (4*′*-CH_3_).

### Cytotoxicity assay

**Figure 1:**
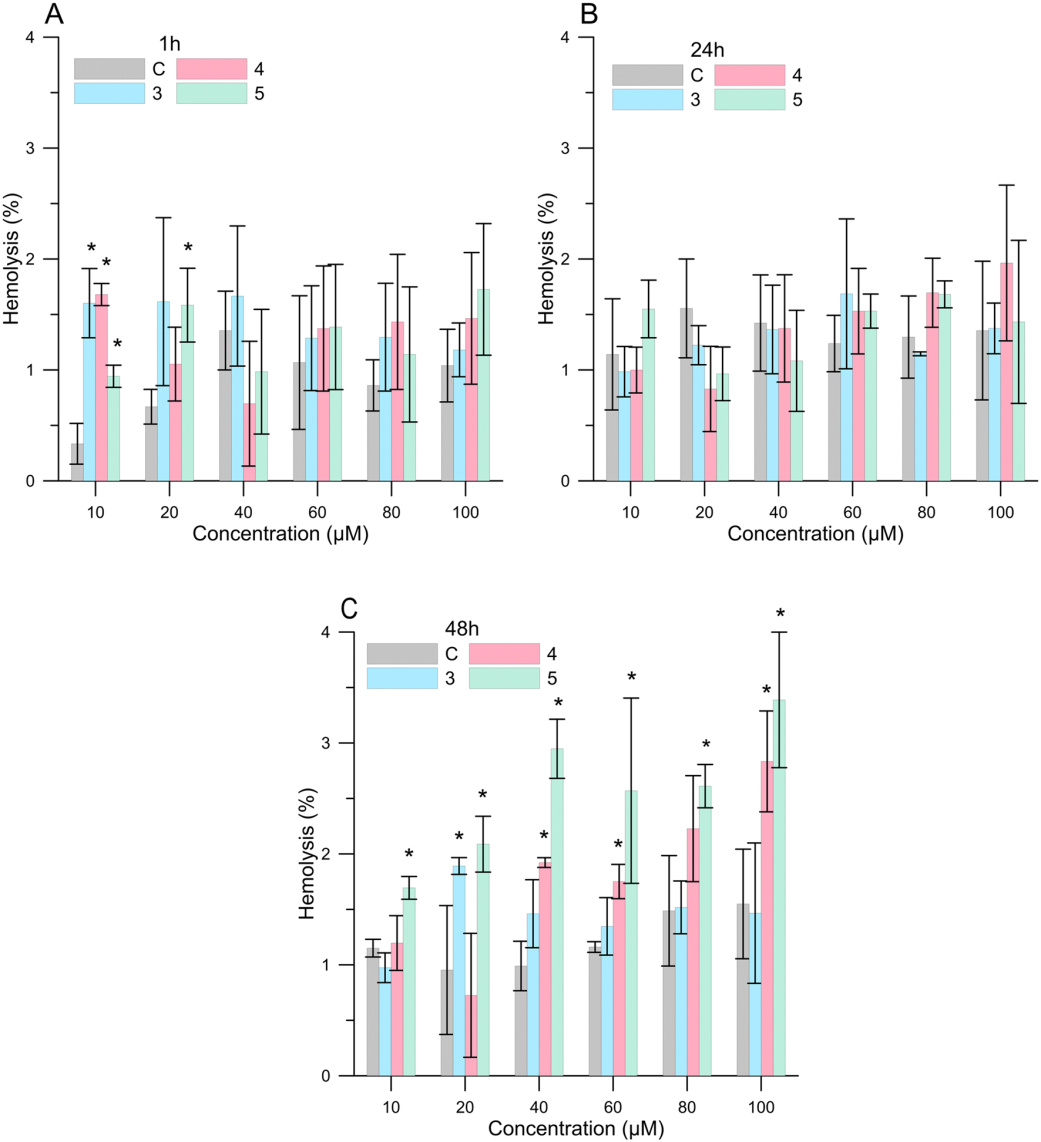
Percentage of hemolysis of RBCs at different concentrations of the compounds (3), (4) and (5) after: (**A**) 1 h, (**B**) 24 h and (**C**) 48 h of incubation.

Cytotoxicity assay provides an evaluation of the effect of different concentrations of compounds on erythrocytes. In our study, we examined cytotoxicity of three compounds namely: 2*′*-hydroxy-4-methylchalcone (**3**), 4*′*-methylflavanone (**4**), and 4*′*-methylflavone (**5**) with respect to red blood cells and after 1, 24 and 48 h of incubation. Compounds were tested at the following concentrations: 10, 20, 40, 60 80 and 100 μM. In Fig. 1, the relationship between the hemolysis (as a percentage) and the concentration of 3, 4 and 5 compounds after different incubation times is shown. The results indicated that the % of hemolysis for all compounds was close to the control after 1 and 24 h of incubation. However, after 48 h of incubation, a slight increase in the percentage of hemolysis for erythrocytes modified by flavonoids was observed, in particular for compound (**5)**. The increase observed in the amount of hemolyzed cells in the presence of compounds (**5**) was negligible and did not exceed 3.4% (± 0.61) for 100 μM concentration. This means that the tested compounds are not toxic.The compounds are considered to be slightly toxic when they achieve hemolysis above 10%, toxic when hemolysis is above 50%, and highly toxic with hemolysis exceeding 90%^19, 20^. These results are compatible with the hemolytic activity studies conducted by Cyboran et al. for green tea supplement (GP), epigallocatechin-3-gallate (EGCG), and gallic acid (GA), measured after 1, 2 and 3 h of incubation^21^. Only EGCG after 3 h of incubation caused a slight increase of hemolysis to 5%^21^. Pawlikowska-Pawlęga et al. showed that quercetin does not show a hemolytic effect on human erythoryce suspended in isotonic solution^22^. Moreover, it causes a protective effect against hemolysis in hypotonic solutions. Furthermore, extracts of buckwheat or saskatoon, whose main ingredients are compounds from the group of flavonoids, does not cause cytotoxic effect^2,23^. Interesting research was also presented by Suwalski et al. who showed that, on the one hand, the significant protection of *Ungi molinae* aqueous extract in low concentrations is responsible for a considerable reduction in the deleterious capacity of hypochlorous acid (HClO) to induce hemolysis in red blood cells^24^. On the other hand, with increasing concentration of the extract (5 × 10^–4^, 0.5 and 1 mM GAE), the percentage of hemolysis increased.

Taking everything into account, it can be concluded that new compounds from the group of flavonoids do not cause hemolysis of RBCs and, therefore, do not have any toxic effect.

### Biophysics research

#### Fluorimetric method

**Figure 2:**
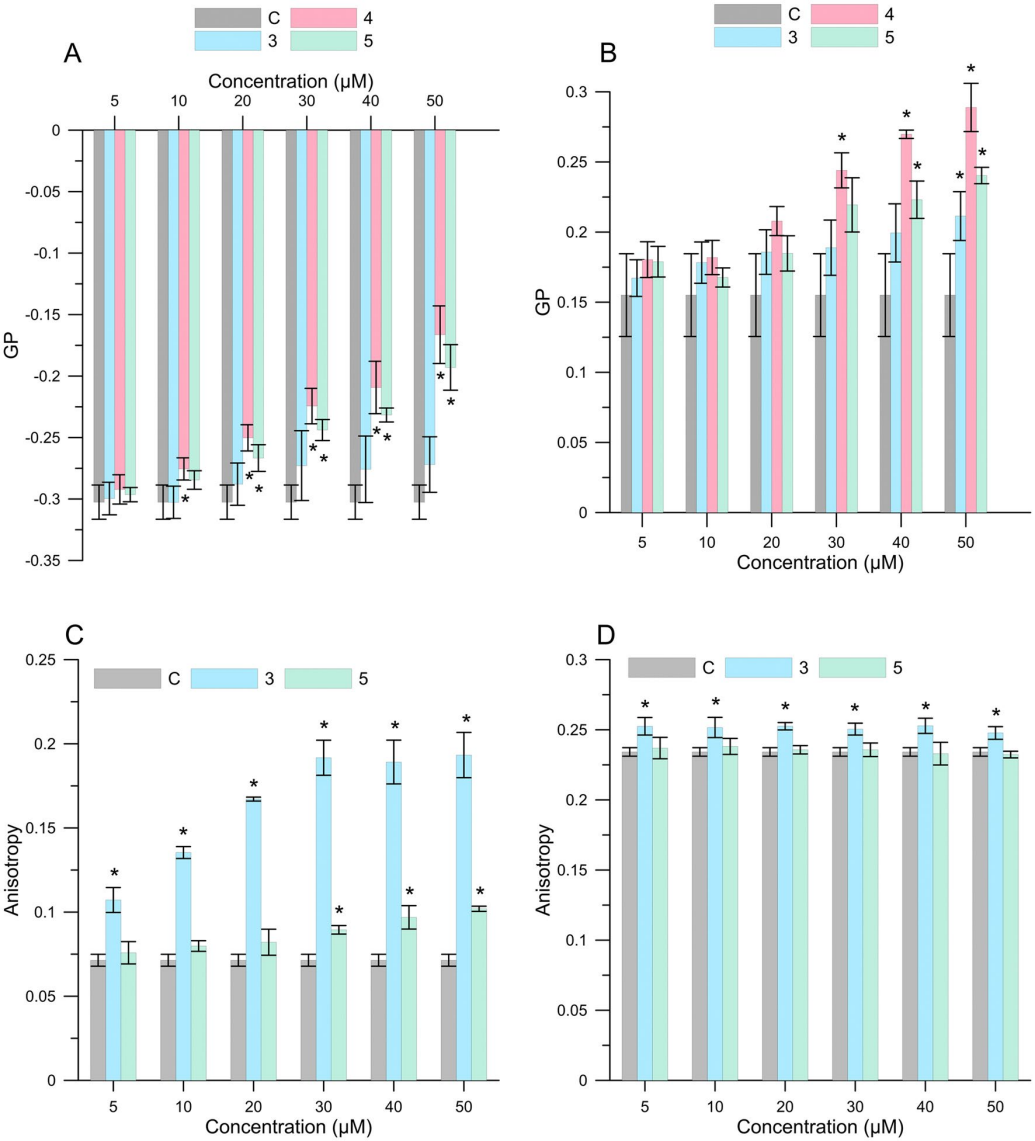
GP of Laurdan probe for (**A**) PC and (**B**) RBCMs membranes in the presence of compounds (3), (4) and (5). Anisotropy fluorescence of DPH probe for (**C**) PC and (**D**) RBCMs membranes in the presence of compounds (3), and (5). Means labeled with asterisk (*) are significantly (*p* < 0.05) different from control. PC- phosphatidylcholine liposomes, RBCMs-red blood cells membranes. C—control, (3) 2*′*-hydroxy-4-methylchalcone(4) 4*′*-methylflavanone, (5) 4*′*-methylflavone.

In order to determine the effect of the flavonoid compounds on the properties of RBCMs and liposomes, two fluorescent probes, namely Laurdan and DPH, were used. The first of them provided information about packing order in the polar group of lipids and hydration in the hydrophilic area of the bilayers. These changes were determined on the basis of the parameter of general polarization (GP). Figure 2A,B shows the values of GP for control (PC or RBCMs) and membranes modified affected by the tested compounds. The results showed that although all compounds affect the hydrophilic region of the membrane, they exert their effect to a different extent. Furthermore, the compounds cause an increase in GP comparing to control. The value of GP was different depending on the membrane used, namely, there was a positive value for RBCMs (Fig. 2B) and negative for PC (Fig. 2A). This is due to the structure of membrane. Whereas a more ordered structure is typical for RBCMs, liquid, disordered structures, are typical for liposomes^1^. The largest increase of GP in both types of membranes resulted compound (**4**) **(**statistically significant changes in the case of PC was in the concentration range of 10–50 µM, and in the case of RBCMs in the concentration range of 30–50 µM, *p* < 0.05**)** and the smallest compound (**3**). The obtained results indicate an increase in the order in the hydrophilic region of the membrane, and thus a decrease in water content in this region. The influence of flavonoids on the increase in the packing order in the polar head group of lipids was also confirmed by numerous authors. According to Veiko et al. naringenin (which is a representative of flavonoids) showes a dose- dependently increased in the order of lipid packing and decreased the hydration^25^. Also (-)-epicatechin or an extract obtained from Japanese quince caused an increase in the GP in the case of PC membrane which may result of hydrogen bonds between the hydroxyl groups of the flavonoid and the polar head groups of phospholipids^1,8^.

The second probe used in the research, which has an affinity for the hydrocarbon chains in the membrane, was DPH. On the basis of changes in fluorescence anisotropy of a DPH probe under the influence of a tested substance, information about changes in fluidity of the membrane which occurred in the hydrophobic region was obtained. Figure 2C,D shows the values of anisotropy for control (pure membranes) and membranes with the addition of compounds (**3**) and (**5**). 4*′*-Methylflavanone had its own fluorescence in the region of the emission spectrum of the DPH probe, therefore it was not possible to determine anisotropy changes in this compound. A plot of the dependence of the fluorescence intensity for compound (**4**) on its concentration was included in supplementary materials (Fig. 41). In the case of compound (**3**) it was observed that it cause an increase in fluorescence anisotropy regardless of the used membranes, across the range of concentrations (statistically significant changes, *p* < 0.05). 4*′*-Methylflavone caused an increase in fluorescence anisotropy only in relation to liposome membranes (Fig. 2C), in the case of RBCMs the value of anisotropy was at the control level (Fig. 2D). An increase in the value of anisotropy indicates a decrease in fluidity of the membrane, and thus an increase in stiffness in the hydrophobic region.

We can assume, that the enormous increase in stiffness in the hydrophobic area (after adding 2*′*-hydroxy-4-methylchalcone) is due to the strength of the hydrophobic interactions between these compound and phospholipids and also their ability to form intramolecular and intermolecular hydrogen bonds^26^. The weaker impact of compounds on the RBMCs might be the result of a different construction of this membrane in comparison to model membranes of liposomes. RBCMs have a more complicated structure, due to the presence of sphingomyelin, cholesterol or the possible presence of cytoskeleton proteins^27^, in comparison to the membranes of liposome composed only of phospholipids. Our previous studies on the interaction of the polyphenol extract from Japanese quince and its important constituents in relation to the RBCMs and liposomes with PC led us to a similar conclusion^1^. Arora et al.showed that flavonoids (naringenin and rutin) and isoflavonoids (genistein) penetrate into the hydrophobic core of the membrane and stabilize it through a decrease in lipid fluidity^26^. Van Dijk et al. showed that (planar) flavonols have a tendency to exhibit a substantially higher affinity for the phospholipid bilayer of the membrane vesicles when compared with (non-planar) flavanones^28^. Wesołowska et al. reached a similar conclusion while analyzing geometry-optimized structures of chalcone (xanthohumol and isoxanthohumol) and flavanones (8-prenylnaringenin)^29^. It was discovered that a chalcone unit in xanthohumol shows a tendency to be planar, in contrast to flavonoid backbone. It may explain that xanthohumol is able to the deeper penetration of towards the hydrophobic region of the bilayer. A linear shape of molecule would also facilitate its interaction with the acyl chains of lipids. Differences in the structure of individual groups of flavonoids as well as the presence of additional functional groups may affect the type of interactions between flavonoids and the lipid bilayer^30^. According to Schoefer et al. the reason behind the lower fluorescence quench of a DPH probe is seen in the difference in structure flavanones and chalcones^31^. To compare, chalcones possess one double bond in lieu of the ring C and one oxo group which together form the π-conjugated system throughout the whole molecule, while lack of a double bond in the ring C of flavanones restricts the size of conjugated double bond system within their molecules^29^.

#### FTIR method

**Figure 3:**
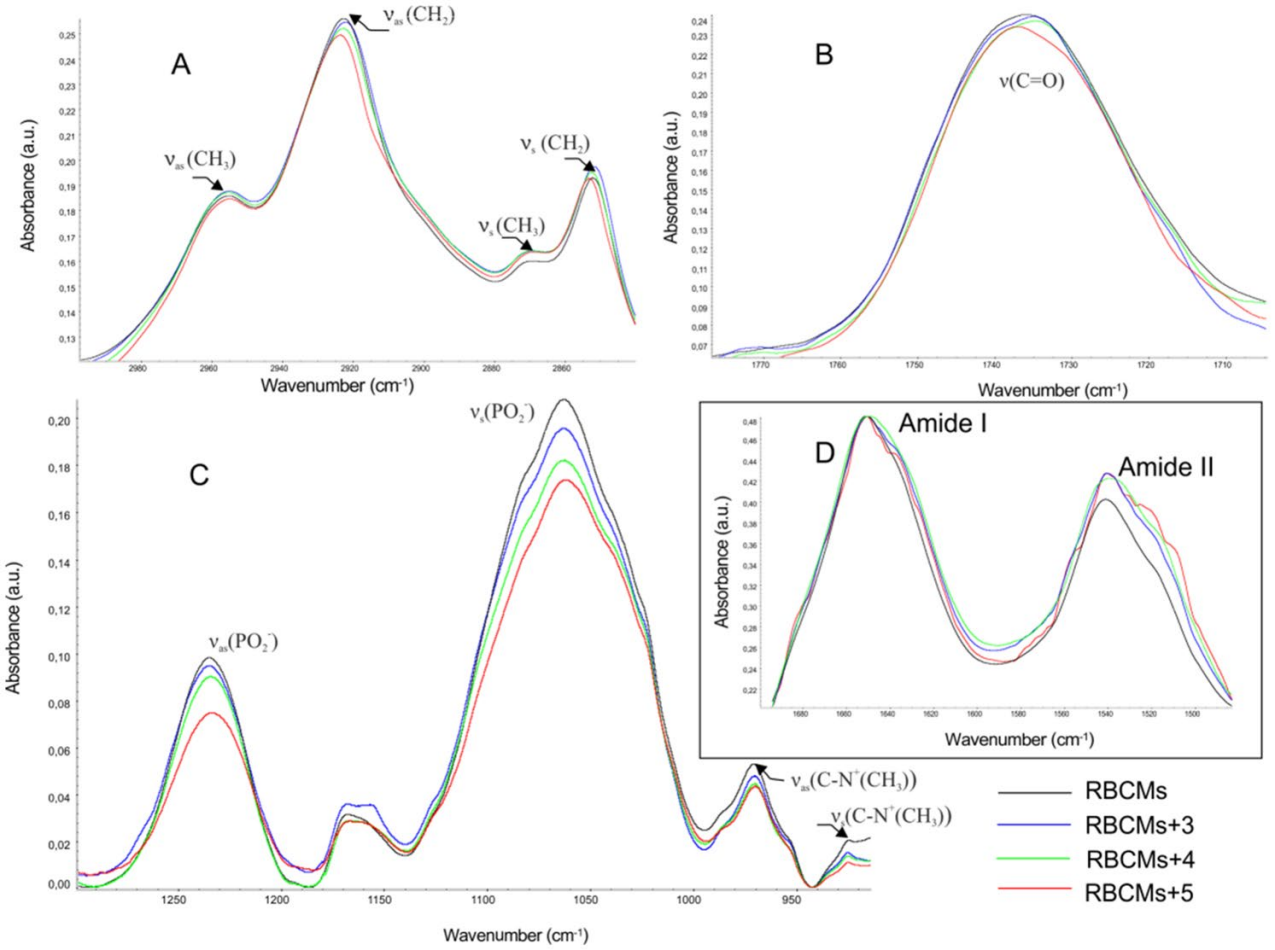
FTIR spectra of RBCMs and of RBCMs modified with flavonoids, concentration of compounds 50 µM; (**A**) C–H stretching regions, (**B**) carbonyl band, (**C**) phosphate and choline band of lipids, (**D**) amide bands.

**Figure 4:**
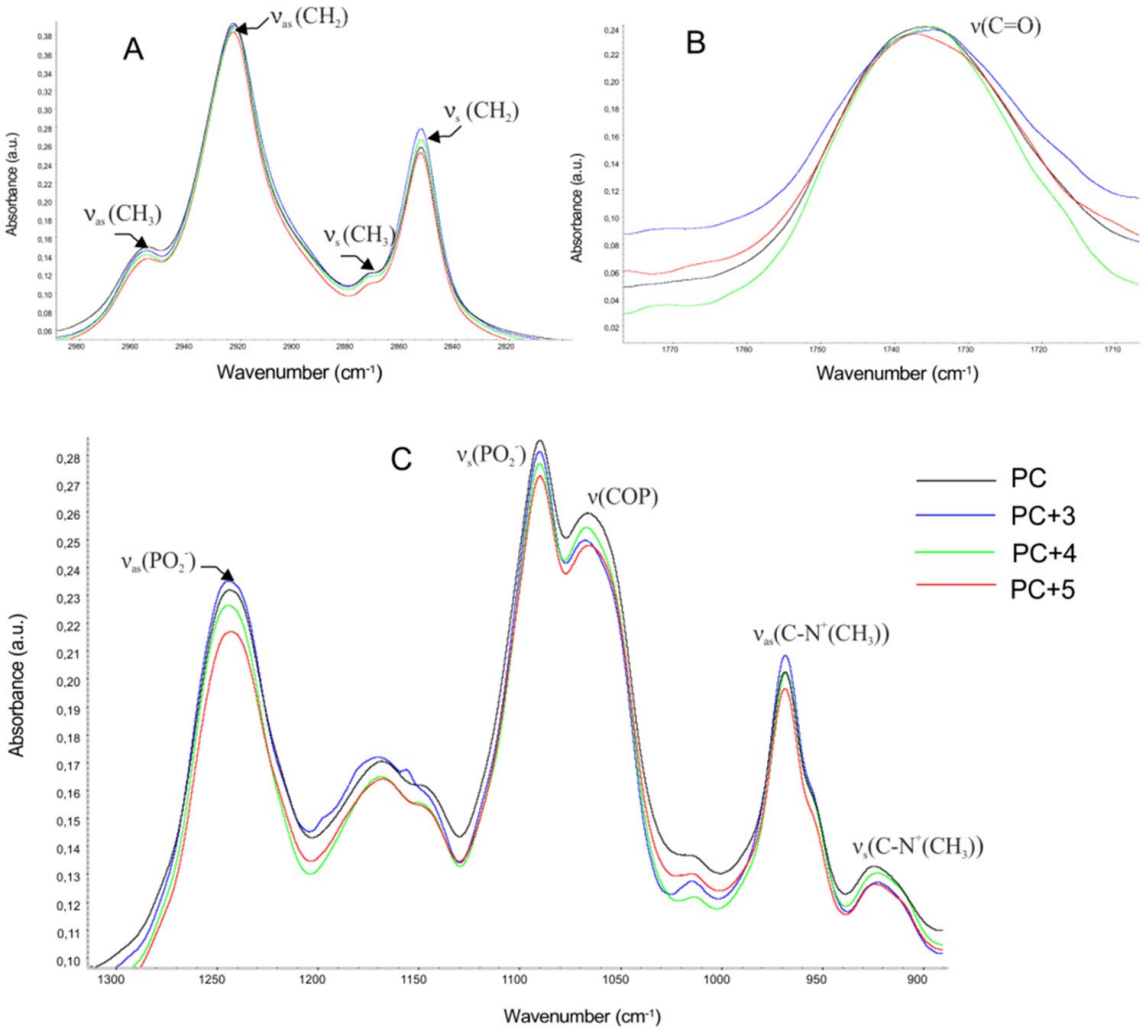
FTIR spectra of PC liposome’s and of PC modified with flavonoids, concentration of compounds 50 µM; (**A**) C–H stretching regions, (**B**) carbonyl band, (**C**) phosphate and choline bands of lipids.

In order to collect more detailed information concerning molecular mechanisms of interaction between three flavonoids and biomolecules, the lipid–protein membrane (RBCMs) and lipid model membrane (PC) were analyzed using ATR-FTIR technique. All measurements were performed at 310 K for two selected concentrations of polyphenols, namely 25 and 50 µM. The most significant frequencies of lipids for RBCMs and RBCMs in the presence of compounds are shown in Table S1. Figures 3 and 4 summarize the most relevant areas of the infrared spectra of the samples composed of pure RBCMs (or PC) and with the 50 μM addition of compounds. The main vibration bands of lipids within the range from 3000 to 900 cm^−1^ (Figs. 3 and 4A–C) as well as these created by proteins of erythrocyte membranes (Fig. 3D) were analyzed. The results of this study prove that the tested flavonoids have an impact on biophysical properties of two model membranes. Figures 3A and 4A present a 2800–3000 cm^−1^ range which is associated with lipid vibrations of methylene and methyl groups of hydrophobic chains. The maximums of asymmetrical and symmetrical stretching vibrations of methylene groups are located at about 2922 cm^−1^ (*v*_as_(CH_2_)) and 2852 cm^−1^ (*v*_s_(CH_2_)), respectively. The presence of flavonoids resulted in a small change in the frequency of CH_2_ group signals (asymmetrical and symmetrical stretching vibrations). These changes vary depending on the structure of the relationship. The presence of chalcone (**3**) causes a slight shift of the wavenumbers towards lower value. In contrast to flavones, an increase in the wavenumber to about 2924 cm^−1^ and 2853 cm^−1^ for ν_as_(CH_2_) and ν_s_(CH_2_) was observed respectively (Fig. 3A, Table S1). A slight difference in the impact of the tested compound on the hydrophobic region of the membrane was observed depending on the membrane type (RBCMs, PC), namely more significant differences between individual compounds were observed for RBCMs. On the basis of the vibrational frequency of methyl groups, one can conclude that the presence of flavonoids causes a slight change in the fluidity of the hydrophobic region of the lipid bilayer—leading it to stiffness in the case of chalcone. Moreover, fluorimetric studies showed that this compound causes an increase in fluorescence anisotropy regardless of the membrane model and with changes being significant for liposomes.

The next band analyzed here was a band of carbonyl group vibration within the range of 1750–1700 cm^−1^. This is the interfacial region of the lipid bilayer and the values of wave numbers which correspond to *v*(C=O) vibration strictly depend on the content of water molecules^32^. The subcomponents of the band of the ester group at higher wave numbers correspond to the C=O group, interacting with a smaller number of water molecules. The band shift towards lower wavenumbers comes from the vibrations of the group involved in a larger number of hydrogen bonds with polar water molecules. In both RBCMs and PC membranes all tested compounds, especially flavanone (**4**), caused a slight shift of vibration toward lower wavenumbers (Figs. 3B, 4B, Table S1).

An interaction between compounds and the head group of lipids was monitored by analyzing the PO_2-_asymmetric and symmetric double stretching band and the band which represents the C–N^+^(CH_3_) stretching band. The asymmetric vibration bands (*v*_as_(PO_2_^−^), 1255–1225 cm^−1^) are extremely prone to changes in the polarity of the environment as well as the possibility of interaction between hydrogen bonds and water, whereas the symmetrical vibration (*v*_s_(PO_2_^−^), 1095–1060 cm^−1^) mainly reflects changes in the conformation of the phosphate fragment C–O–P–O–C^33^. In the case of symmetrical vibration after adding compounds, only slight changes were observed for both RBCMs and PC membranes (Figs. 3C, 4C). For example, for PC, the maximum for *v*(COP) changed from 1066.33 cm^−1^ for pure PC to 1067.79 cm^−1^ in the presence of chalcone (50 μM). During hydration, the maximum of vibrational band for *v*_as_(PO_2_^−^) shifted to lower wavenumber values, which was probably due to increasing interaction between water molecules and phosphate groups^34^. Flavonoids slightly changed the maximum of asymmetric vibration in the case of phosphate group, particularly of flavone, shifting the maximum of vibrations *v*_as_(PO_2_^−^) for phosphate and going towards lower values of wavenumbers (Figs. 3C, 4C). A decrease in the wavenumber of this band indicates that the phosphate polar head group is located in a more polar environment in which the hydration increased. Similar changes were also observed for these membrane embedded compounds which have OH groups in their structure and which might also form hydrogen bonds with the phosphoric group lipids, e.g. quercetin, genistein, flavanones^29,35^. As Cieślik-Boczula et al. suggest the wavenumber shift for *v*_as_(PO_2_^−^) to lower wavenumber region, which was observed in a genistein-doped phosphatidylocholine liposomes, is induced by the interaction between hydroxyl groups of genistein and proton acceptor PO_2_^−^ groups of phosphatidylocholine^35^.

The vibration stretching bands of the choline group, which form the outermost part of the membrane, occur in about 970 cm^−1^ for ν_as_(C–N^+^(CH_3_) and about 920 cm^−1^ for ν_s_(C–N^+^(CH_3_)^34^. In both RBCMs and PC membranes the presence of flavonoids induced a slight change in this band (Figs. 3C, 4C) namely for pure PC the maximum was reached at ν_s_(C–N^+^(CH_3_) = 924.51 cm^−1^ and for chalcone and flavanone at ν_s_(C–N^+^(CH_3_) = 922.80 cm^−1^. Taking into account these results, the conclusion is that all flavonoids affect the hydrophilic region of membranes.

Additionally, the bands originating from two proteins, namely amide I and amide II are detected in RBCMs membrane (Fig. 3D). Amide I is located within the range of 1610–1695 cm^–1^ and amide II appears within the range of 1480–1575 cm^−1^. Amide I and amide II is used for determining the type of protein secondary structure^20,36^. An analysis demonstrated that chalcone, flavanone, and flavones are able to shift the maximum of both amide I and amide II bands, e.g. for pure RBCMs the maximum of amide I is 1652.45 cm^−1^ and for flavanone it is 1649.74 cm^−1^. These changes in the amide bands may suggest that all investigated compounds slightly affect the structure of proteins present in the RBCMs membrane.

The FTIR results show that the tested flavonoids interact with the lipid bilayers. The obtained results confirm the conclusion stemming from the fluorimetric methods that the tested flavonoids influence on the order of the erythrocyte membrane lipid bilayer.

#### Binding to human serum albumin

The study of flavonoid–protein interactions seems to be at the center of academic interests. Although it is safe to state that both the mode of interaction and detailed binding mechanism between flavonoid compounds and proteins at molecule level are becoming better and better understood^37–39^, in our research, a particular attention was given to HSA fluorescence quenching which was induced by newly received compounds i.e. 2′-hydroxy-4-methylchalcone, 4′-methylflavanone and 4′-methylflavone.

**Figure 5:**
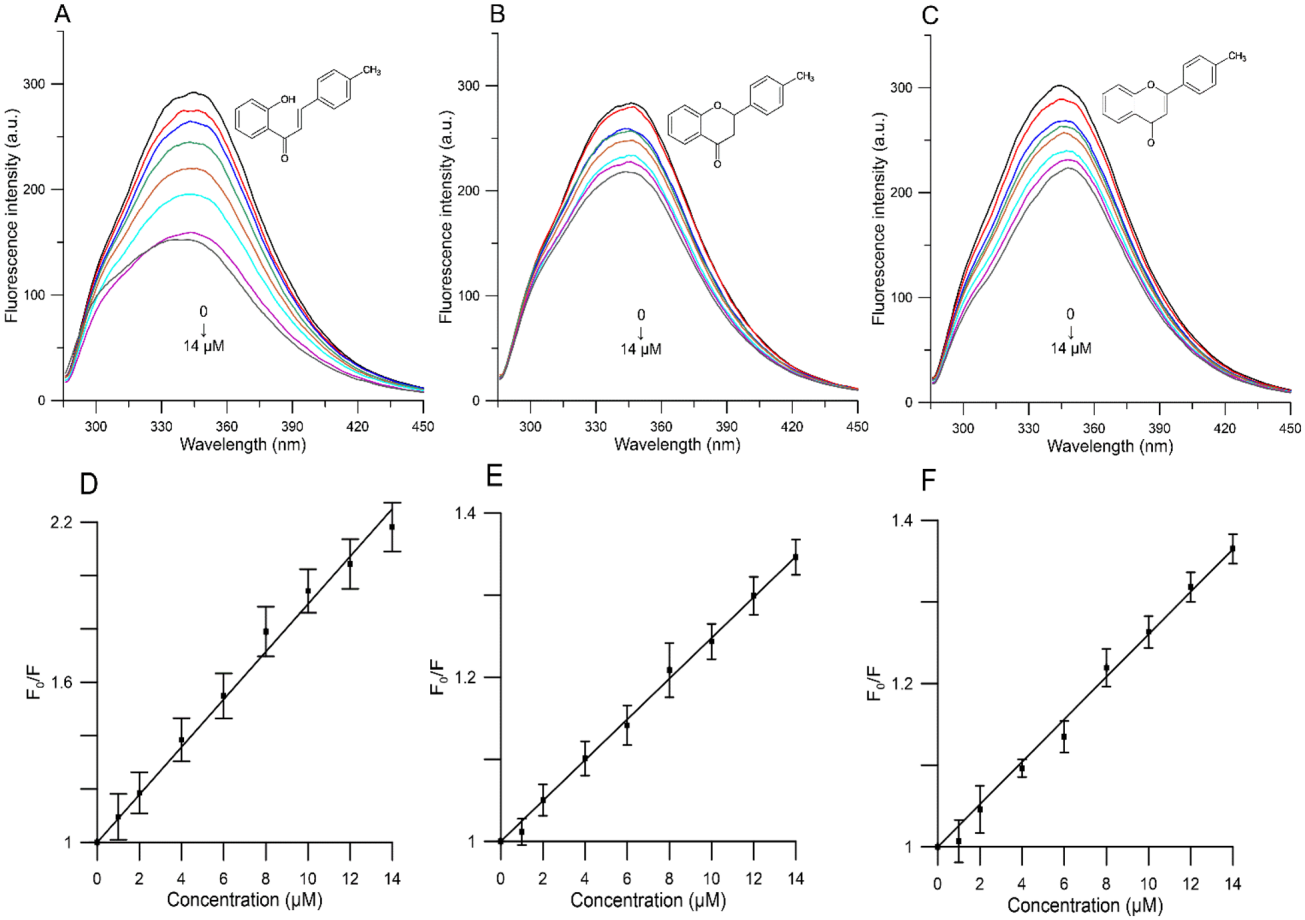
Emission spectra of HSA in the presence of various concentrations of (**A**) 2′-hydroxy-4 methylchalcone, (**B**) 4′-methylflavanone, (**C**) 4′-methylflavone and Stern–Volmer plots of *F*_0_/*F* against concentration of (**D**) 2′-hydroxy-4 methylchalcone, (**E**) 4′-methylflavanone and (**F**) 4′-methylflavone. Control is marked black and consecutive spectra of the studied compounds (marked color) are in the following concentrations 1, 2, 4, 6, 8, 10, 12 and 14 µM, λ_ex_ = 280 nm, T = 310 K.

Upon excitation at 280 nm, the human serum albumin emits an intense band at 345 nm. The effect of the studied compounds on HSA fluorescence intensity at 310 K is shown in Fig. 5. As one may notice, with the addition of compounds (**3**) (Fig. 5A), (**4**) (Fig. 5B) and (**5**) (Fig. 5C) to HSA, the fluorescence intensity decreases progressively with an increase in compounds concentration. This suggests that all compounds are able to interact with HSA and quench its intrinsic fluorescence. 2′-Hydroxy-4-methylchalcone induced the strongest decrease in the emission of HSA, followed by 4′-methylflavone and 4′-methylflavanone. To confirm the quenching mechanism, we analyzed the fluorescence data at different temperatures (295, 300, 305, 310 K) using the Stern–Volmer Eq. ^40^:

1 $$\frac{{F_{0} }}{F} = 1 + K_{q} \tau_{0} \left[ Q \right] = 1 + K_{SV} \left[ Q \right]$$

where: *F*_*0*_ and *F* refer to HSA fluorescence intensities before and after adding quencher, *K*_*q*_ refers to a bimolecular quenching constant, τ_0_ refers to quenching rate constant and average lifetime (10^–8^ s^–1^), [*Q*] refers to the quencher concentration, and *K*_*SV*_ refers to the Stern–Volmer quenching constant (*K*_*q*_ = *K*_*SV*_/*τ*_*0*_).

Quenching is defined as a decrease in fluorescence and is propelled by a dynamic and static quenching mechanism. Whereas quenching constants decrease with increasing temperature in static quenching, the inverse effect is observed in dynamic quenching. This may be explained by an increase in diffusion coefficients and a decrease in stability of the complex at higher temperatures^41^. The fluorescence quenching data at 310 K was presented as Stern–Volmer plot in Fig. 5D–F. The calculated quenching constants *K*_*sv*_ at the corresponding temperature are shown in Table 1. The *K*_*SV*_ values decreased as the temperature increased which indicates that the quenching mechanism of interaction between all compounds and HSA was initiated by static collision. Additionally, the values of bimolecular quenching rate constants for all compounds were obtained about 10^12^ M^− 1^ s^− 1^ (Table 1). There are two orders of magnitude larger than the value of a diffusion limited rate constant of a biomolecule (1.0 ‧ 10^10^ M^−1^ s^−1^)^42^, which seems to confirm the view that the possible mechanism of fluorescence quenching is static. The obtained values of *K*_*sv*_ for compounds (**3**), (**4**), and (**5**) in our analysis are comparable with those present in literature for chalcones^43^, flavone^44^ and flavanone^45^ that are structurally similar to our studied compounds.

**Table 1:** Quenching (*K*_*sv*_) and binding (*K*_*b*_) and bimolecular quenching (*K*_*q*_) constants, number of binding sites (*n*) and thermodynamic parameters (*ΔG, ΔH, ΔS*), of the 2*′*-hydroxy-4-methylchalcone, 4*′*-methylflavanone, and 4*′*-methylflavone and human serum albumin at different temperatures.

Compound	*T* (K)	*K*_*sv*_ (10^4^ M^−1^)	*Kq* (10^12^ M^−1^ s^−1^)	*K*_*b*_ (10^4^ M^−1^)	*n*	∆G (kJ/M)	*∆H* (kJ/M)	∆S (J/(M K))
2′-Hydroxy-4-methylchalcone (**3**)	295	7.03 ± 0.18	7.03 ± 0.18	189.35 ± 0.92	1.27 ± 0.14	− 37.21 ± 1.55	− 77.83 ± 7.06	− 138.67 ± 16.82
	300	6.50 ± 0.20	6.50 ± 0.20	120.01 ± 14.63	1.21 ± 0.14	− 35.86 ± 1.60		
	305	6.22 ± 0.05	6.22 ± 0.05	42.27 ± 3.31	1.16 ± 0.13	− 31.87 ± 2.55		
	310	5.70 ± 0.26	5.70 ± 0.26	15.99 ± 1.00	1.10 ± 0.11	− 33.37 ± 2.21		
4′-Methylflavanone (**4**)	295	3.20 ± 0.08	3.20 ± 0.08	3.89 ± 0.37	1.23 ± 0.38	− 25.91 ± 0.23	− 156.26 ± 35.29	− 577.51 ± 74.84
	300	3.07 ± 0.02	3.07 ± 0.02	1.63 ± 0.86	1.13 ± 0.33	− 23.99 ± 1.38		
	305	2.90 ± 0.02	2.90 ± 0.02	1.65 ± 1.58	1.02 ± 0.35	− 20.94 ± 1.19		
	310	2.47 ± 0.02	2.47 ± 0.02	0.91 ± 0.28	0.94 ± 0.28	− 19.58 ± 0.88		
4′-Methylflavone (**5**)	295	3.10 ± 0.31	3.10 ± 0.31	6.13 ± 0.43	1.03 ± 0.13	− 27.03 ± 0.17	− 40.37 ± 8.02	− 53.98 ± 13.47
	300	2.86 ± 0.43	2.86 ± 0.43	5.05 ± 0.13	1.02 ± 0.04	− 26.75 ± 0.44		
	305	2.75 ± 0.46	2.75 ± 0.46	4.12 ± 0.24	0.97 ± 0.10	− 26.28 ± 1.09		
	310	2.48 ± 0.17	2.48 ± 0.17	2.42 ± 0.09	0.93 ± 0.11	− 24.62 ± 2.06		

**Figure 6:**
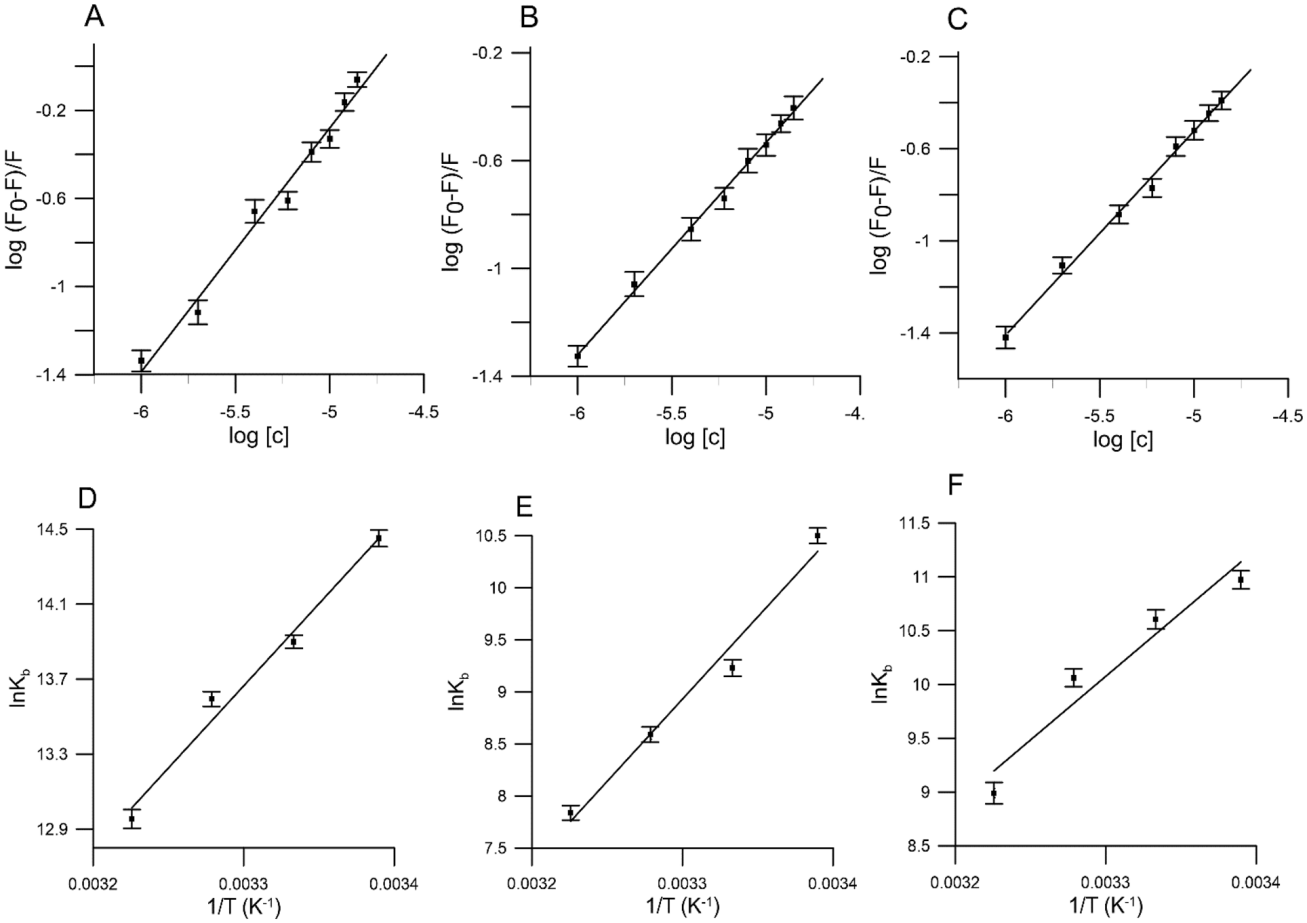
The plots of log(*F*_0_ − *F*)/*F versus* log[*c*] of (**A**) 2′-hydroxy-4 methyl chalcone, (**B**) 4′-methylflavanone, (**C**) 4′-methylflavone at 310 K and van't Hoff plot for temperature dependence of *K*_*b*_ obtained from HSA fluorescence quenching by (**D**) 2′-hydroxy-4 methyl chalcone, (**E**) 4′-methylflavanone and (**F**) 4′-methylflavone.

In order to obtain information about the association between HSA and the studied compounds, the binding constant (*K*_*b*_) and the number of binding sites (*n*) were calculated employing a modified Stern–Volmer equation:

2 $$log\left( {\frac{{F_{0} - F}}{F}} \right) = logK_{b} + nlog\left[ Q \right]$$

where, *F*_0_ and *F* indicate the magnitude of fluorescence intensity in the absence and presence of quencher (studied compounds), *K*_*b*_ is the binding constant and *n* is the number of binding sites. From linear plot of log (*F*_*0*_* − F)/F* versus log [*Q*], the values of *K*_*b*_ for all compounds was obtained from the intercept (Fig. 6A–C).The results of the study based on the tested compounds at four different temperatures are presented in Table 1.

As it is illustrated in the Table 1, the *K*_*b*_ values decreased with increasing temperature causing a decrease in the binding affinity of all compounds to HSA. The association chalcone: HSA, flavone: HSA and flavanone: HSA are within the range of 10^4^ M^− 1^, showing a good interaction between all of these compounds and human serum albumin^46^. The *K*_*b*_ values ranked in the following order: chalcone > flavone > flavanone. Moreover, we noticed that the number of binding sites between compounds: (**3**), (**4)**, (**5**) and HSA at four different temperatures is approximately equal 1 (Table 1) which indicates that there is only one compound molecule which is bound to one albumin molecule.

In general, chalcones have the highest binding constants and are followed by both flavones and flavanones which have the lowest values of binding constants. These results are in line with another data^43,47^and our previously published studies^48^. Moreover, in silico and in vitro studies validated the moderate binding of chalcone with BSA^49,50^. Molecular docking and molecular dynamic simulation studies suggested the stabile binding of chalcone with the BSA microenvironment^49^. Major structural differences between the compounds used in our study are related to the ring C which correlates with binding to HSA. Flavones, unlike flavanones, have a double bond between positions 2 and 3 and are saturated while chalcones do not possess the ring C in the basic flavonoid skeleton. Studies conducted by López-Yerena et al. showed that flavanones have extremely low nucleophilicity as well as a high negative partial charge, while flavones have medium to high nucleophilicity and a medium negative partial charge^51^. These hydrophobic compounds display stronger binding a nities for HSA (higher binding constants) than the other flavonoids^47,51,52^. The possible explanation is that hydrogenation of the unsaturated double bond of flavone to flavanones modifies the ring C from a planar to a twisted structure, while simultaneously reduce polarity as a result they may have less penetrated into the hydrophobic pockets in the HSA^38^. Planar structure of flavone seems to play a role in binding with HSA in hydrophobic pockets in albumin^38,47^. Another study has found that the hydroxyl group O-methylation reaction enhanced hydrophobicity and hydrophobic interactions increasing affinity for HSA^37,45^.

In order to precisely describe the key forces observed between the studied compounds and HSA, the thermodynamic parameters were analyzed. The main interaction forces between molecules and proteins include: van der Waals, electrostatic, hydrophobic interactions and hydrogen bonds. The values of enthalpy change (*ΔH*) and entropy change (*ΔS)* were calculated using van't Hoff equation:

3 $$lnK_{b} = \frac{ - \Delta H}{{RT}} + \frac{\Delta S}{R}$$

The free-energy change *ΔG* is estimated on the basis of the following interaction:

4 $$\Delta G = \Delta H - T\Delta S = - RTlnK_{b}$$

where: *ΔH, ΔG* and *ΔS* refer to enthalpy change, free enthalpy change, and entropy change. *R* refers to the gas constant 8.314 J mol^−1^ K^−1^.

On the basis of the binding constants of the analyzed compounds to HSA at four temperatures, the thermodynamic parameters *ΔH* and *ΔS* for all compounds were calculated on the basis of a relationship between ln *K*_*b*_ vs. 1/T (Fig. 6D–F). The values of *ΔH*, *ΔG* and *ΔS* for 2′-hydroxy-4-methylchalcone, 4′-methylflavanone and 4′-methylflavone at different temperatures are shown in Table 1.

Ross and Subramanian describe the sign of thermodynamic parameter as associated with various types of interaction that may be observed during the protein association processes^53^. In this sense, when *ΔH* < 0 and *ΔS* < 0, van der Waals interactions and hydrogen bonds are dominant in the reaction, when ΔH > 0 and *ΔS* > 0, hydrophobic interactions are dominant over the binding process and when *ΔH* < 0 and ΔS > 0, electrostatic force is dominant in the interaction.

As shown in Table 1, the negative values of *ΔH* and *ΔS* indicate that interactions between HSA and compounds (**3**), (**4)** and (**5**) are primarily driven by van der Waals forces and hydrogen bonding. The negative values of *ΔH* and *ΔS* also suggest that binding interactions are mostly enthalpy driven^54^. Negative values of the Gibbs free energy change for all compounds analyzed here are in accord with the spontaneity of their binding process to albumin.

## Conclusions

In this study three methylated flavonoids which belong to different classes, i.e. chalcone, flavanone and flavone were obtained. We showed that the compounds (**3**), (**4**) and (**5**) do not have a negative impact on the membranes of blood cells which may be an indicator of their non-toxicity to organism. The fluorimetric method showed that all compounds used here affect the membrane hydrophilic and hydrophobic regions, but they exert their effect to a different extent. The most significant impact on hydrophilic region in both types of membranes was caused by 4′-methylflavanone and the least significant was caused by 2′-hydroxy-4-methylchalcone. Furthermore, 2′-hydroxy-4-methylchalcone had the greatest impact on the hydrophobic area of the membrane. The results were confirmed by the FTIR technique. The experimental data showed that all the compounds interacted with HSA and quenched its intrinsic fluorescence in a way of a static mechanism which was caused by a complex formation. Structural-binding affinity relationships showed that the fluorescence quenching of HSA by 2′-hydroxy-4-methylchalcone was stronger than that observed in the case of 4′-methylflavanone and 4′-methylflavone.

Our preliminary results are the basis for further research on the biological activity and pharmacological efficacy of 4′-methylflavonoids as potential drugs. In the next step of our research, the obtained compounds will be glycosylated using enzyme systems of entomopathogenic fungi, in order to further improve their bioavailability. Afterwards, they will be tested in a similar way as presented in this paper, to compare results for flavonoid aglycons and their glycosides.

## Materials and methods

### Materials

The pig blood for testing came from the Faculty of Veterinary Medicine of the Wrocław University of Environmental and Life Sciences. The material for testing was obtained during the standard diagnostic procedure.

Human serum albumin (lyophilized powder, essentially fatty acid free), 2′-hydroxyacetophenone, 4-methylbenzaldehyde, deuterium oxide (D_2_O), egg yolk phosphatidylcholine (PC) were purchased from Sigma–Aldrich. The probes1,6-diphenyl-1,3,5-hexatriene (DPH) and Laurdan were purchased from Molecular Probes (Eugene, OR, USA).

### Synthesis

HPLC analyses were performed on a Dionex Ultimate 3000 instrument (Thermo Fisher Scientfic, Waltham, MA, USA) with a diode array detector using pre-column and an analytical ODS 2 column (4.6 × 250 mm, Waters, Milford, MA, USA). The mobile phase was a mixture of 0.1% aqueous formic acid v/v (A) and 0.1% formic acid in acetonitrile v/v (B). The gradient program was as follows: initial conditions—32.5% B in A, 4 min—40% B in A, 8 min—40% B in A, 10 min—45% B in A, 15 min—95% B in A, 18 min—95% B in A, 19 min—32.5% B in A, 23 min—32.5% B in A. The flow rate was 1 mL/min, the injection volume was 5 µL, and detection wavelength 280 nm. Details have been described in our earlier work^18^.

NMR analyses (^1^H-NMR, ^13^C-NMR, COSY, HSQC, HMBC) were performed using a DRX Avance™ 600 MHz NMR spectrometer (Bruker, Billerica, MA, USA). The solvent was deuterated acetone. Optical rotation was measured with the digital polarimeter P-2000-Na (ABL&E-JASCO, Kraków, Poland).

### Liposome preparation

Small unilamellar liposomes (SUVs) composed of phosphatidylcholine (PC) were prepared according to the procedure described ealier by Gabrielska and Oszmiański^55^. The PC were dissolved in chloroform, evaporated to dryness under nitrogen and under vacuum for 1 h. Next, the sample was hydrated and vortexed to obtain of multilamellar vesicles. Then, using a 20 kHz sonicator for 15 min were formed unilamellar liposomes (SUVs). The final concentration of lipids was 0.1 mg/mL^56^.

### Preparation of erythrocytes and their membranes

In the studies used red blood cell membranes (RBCMs) as a simple protein-lipid model of the biological membrane and red blood cells (RBCs) as good model to evaluate the cytotoxicity of compounds by cellular damage measure^2,19,57^. RBCs were prepared according to the procedure described in our earlier work^2,57^. Briefly, fresh blood was taken to a physiological solution of sodium chloride with heparin added, then was centrifuged at 2500 rpm for 3 min at 277 K and other blood components such as the platelets and leukocytes contained in supernatant were disposed. After that erythrocytes were washed with cold phosphate buffered saline isotonic solution. The RBCs prepared in this way were used to isolate their membranes (RBCMs) according to the procedure described in the work^58^ and for cytotoxicity assay.

### Cytotoxicity assay

A cytotoxicity assay is the first test before any investigation of biological activity or interaction the new compounds with biomolecules and membranes. This method, called hemolysis, is based on measurement of the leakage of hemoglobin from RBCs and was described in detail in our earlier work^57^. For erythrocytes at a hematocrit of 1.2% were added compounds at concentration: 10, 20, 40, 60, 80 and 100 μM. Samples thus prepared were incubated for 1, 24 and 48 h at 310 K. After incubation the samples were centrifuged and was determined the hemoglobin concentration in the supernatants using an UV–Vis spectrophotometer (Specord 40, AnalytikJena) at 540 nm wavelength^59^. The percentage of RBCs hemolysis, is counted as absorbance of hemoglobin in tested samples to the absorbance of hemoglobin of totally hemolyzed cells (100%). Total hemolysis samples (100%) were prepared by adding distilled water to the samples.

### Biophysics research

In biophysical studies, the fluorimetric and Fourier Transform Infrared Spectroscopy (FTIR) methods were used to determine the effect of the new compounds on the properties of the biological membranes and to study their interaction with biomolecules. Tested compounds were dissolved in ethanol, therefore in all biophysical studies ethanol was added to each control sample in an amount appropriate to the concentration of the test samples. The concentration ranges were used appropriately selected to the type of methods and biomolecules used.

### Fluorimetric method

For fluorimetric research were used RBCMs a and small liposomes (SUV) as an example of biomolecules. The content of RBCMs in the samples was determined on the basis of protein concentration, which was assayed using the Bradford method^60^, and it was the same in all experiments. The concentration of lipid was 0.1 mg/mL. In this method was used two fluorescent probes like Laurdan and DPH at a concentration of 1 μM. The fluorescent group of Laurdan is located at the level of lipid ester groups^61^ but DPH is located along the axis of the fatty acyl chains of the phospholipids in the bilayer the DPH^1^. The first of them informs us about the packing arrangement in the polar group of lipids based on generalized polarization (GP) and the second about fluidity of the hydrocarbon chains based on the fluorescence anisotropy. Briefly, the mixture contained RBCMs or liposomes and fluorescent probes were suspended in isotonic phosphate solution of pH 7.4 were incubated for 30 min in darkness at room temperature. Next, the mixture was dispensed into cuvettes and the studied compounds were added in the range from 5 to 50 μM. Thus prepared samples were incubated for 1 h at 310 K. After 1 h, measurements were conducted with a fluorimeter (Cary Eclipse, Varian, Palo Alto, CA, USA) at 310 K. The excitation and emission wavelengths were as follows: for DPH λ_ex_ = 360 nm and λ_em_ = 425 nm; and for probe Laurdan λ_ex_ = 360 nm and λ_em_ = 440 and 490 nm.

Fluorescence anisotropy for DPH was calculated using the formula^40^:

5 $$A = \frac{{I_{\parallel } - GI_{ \bot } }}{{I_{\parallel } + 2GI_{ \bot } }}$$

where *I*_*I*I_ and *I*_*⊥*_ are fluorescence intensities observed in directions parallel and perpendicular, respectively, to the polarization plane of the exciting wave. G is an apparatus constant dependent on the emission wavelength.

The generalized polarization (GP) for Laurdan were calculated with the formula^61^:

6 $$GP = \frac{{I_{b} - I_{r} }}{{I_{b} + I_{r} }}$$

where *I*_*b*_ is fluorescence intensity at λ = 440 nm, and *I*_*r*_ is fluorescence intensity at λ = 490 nm. The experiment was performed in six replicates (n = 6).

### FTIR method

FTIR method was used determined the interactions between the compounds and specific functional groups of lipids as choline, carbonyl, phosphate, hydrocarbon chains (in PC and RBCMs) and additionally groups of proteins as amide I and II in RBCMs. Detailed procedures for the preparation of RBCMs and PC are described in ealier. The procedure of sample preparation has been described in our previous papers^3,20,57^. Briefly, after to remove the water the spectrum of the control (RBCMs or liposomes) and tested samples (RBCMs or PC with tested compounds) at concentration 50 and 25 µM was taken. The measurements were performed at 310 K using a Thermo Nicolet 6700 MCT (Thermo Fisher Scientific, Waltham, MA, USA). Next, the dried probes was hydrated in aqueous solution and the measurements was repeated. Each single spectrum was obtained in the range of 700–4000 cm^−1^.

### Binding to HSA

Analysis of interaction of studied compounds with HSA was performed according to the work of Strugała et al.^56^ with minor modifications. Fluorescence spectra were recorded on a fluorimeter (Cary Eclipse, Varian, Palo Alto, CA, USA). The excitation wavelength was set at 280 nm, and the emission spectra were read at 285–460 nm. Stock solution of HSA was prepared in the in a phosphate buffer of pH 7.4. and final concentration was 1.5‧10^–5^ M. The final concentrations of the study compounds was 1, 2, 4, 6, 8, 10, 12 and 14 µM. The fluorescence was measured at four temperatures (295, 300, 305 and 310 K). The experiment was replicated four times (n = 4).

### Statistical analysis

Statistical analysis was performed using the program Statistica 12.0 (StatSoft, Kraków, Poland). The results were analyzed by one-way ANOVA followed by Duncan test. *p* values < 0.05 were considered statistically significant. All the experiments were done in at least four replicates and the results were expressed as means values ± standard deviation (SD).

## Supplementary Information

Supplementary Information.
