# Supplementary material for: Interaction of 4′-methylflavonoids with biological membranes, liposomes, and human albumin
Source: Sci Rep. 2021 Aug 6;11:16003. doi: 10.1038/s41598-021-95430-8 (PMC8346624; doi:10.1038/s41598-021-95430-8)
Supplement: Supplementary file 1 — Supplementary Information. [file 41598_2021_95430_MOESM1_ESM.pdf]

Interaction of 4'-methylflavonoids with biological membranes, liposomes, and human albumin

Aleksandra Włoch[1], Paulina Strugała-Danak[1*], Hanna Pruchnik[1], Agnieszka Krawczyk-Łebek[2], Karolina Szczecka[1], Tomasz Janeczko[2], Edyta Kostrzewa-Susłow[2]

[1]Department of Physics and Biophysics, Wrocław University of Environmental and Life Sciences, C. K. Norwida 25, 50-375 Wrocław, Poland
[2]Department of Chemistry, Wrocław University of Environmental and Life Sciences, C. K. Norwida 25, 50-375 Wrocław, Poland

*email: paulina.strugala@upwr.edu.pl

Supplementary materials

Content

Figure S1. ^{1}H NMR spectrum of 2′-hydroxy-4-methylchalcone (**3**) (Acetone-d_6, 600 MHz)
Figure S2. ^{1}H NMR spectrum of 2′-hydroxy-4-methylchalcone (**3**) (Acetone-d_6, 600 MHz)
Figure S3. ^{13}C NMR spectrum of 2′-hydroxy-4-methylchalcone (**3**) (Acetone-d_6, 151 MHz)
Figure S4. ^{13}C NMR spectrum of 2′-hydroxy-4-methylchalcone (**3**) (Acetone-d_6, 151 MHz)
Figure S5. COSY NMR spectrum of 2′-hydroxy-4-methylchalcone (**3**) (Acetone-d_6, 600 MHz)
Figure S6. COSY NMR spectrum of 2′-hydroxy-4-methylchalcone (**3**) (Acetone-d_6, 600 MHz)
Figure S7. COSY NMR spectrum of 2′-hydroxy-4-methylchalcone (**3**) (Acetone-d_6, 600 MHz)
Figure S8. HSQC NMR spectrum of 2′-hydroxy-4-methylchalcone (**3**) (Acetone-d_6, 151 MHz)
Figure S9. HSQC NMR spectrum of 2′-hydroxy-4-methylchalcone (**3**) (Acetone-d_6, 151 MHz)
Figure S10. HSQC NMR spectrum of 2′-hydroxy-4-methylchalcone (**3**) (Acetone-d_6, 151 MHz)
Figure S11. HMBC NMR spectrum of 2′-hydroxy-4-methylchalcone (**3**) (Acetone-d_6, 151 MHz)
Figure S12. HMBC NMR spectrum of 2′-hydroxy-4-methylchalcone (**3**) (Acetone-d_6, 151 MHz)
Figure S13. HMBC NMR spectrum of 2′-hydroxy-4-methylchalcone (**3**) (Acetone-d_6, 151 MHz)
Figure S14. HMBC NMR spectrum of 2′-hydroxy-4-methylchalcone (**3**) (Acetone-d_6, 151 MHz)
Figure S15. ^{1}H NMR spectrum of 4′-methylflavanone (**4**) (Acetone-d_6, 600 MHz)
Figure S16. ^{1}H NMR spectrum of 4′-methylflavanone (**4**) (Acetone-d_6, 600 MHz)
Figure S17. ^{13}C NMR spectrum of 4′-methylflavanone (**4**) (Acetone-d_6, 151 MHz)
Figure S18. ^{13}C NMR spectrum of 4′-methylflavanone (**4**) (Acetone-d_6, 151 MHz)
Figure S19. COSY NMR spectrum of 4′-methylflavanone (**4**) (Acetone-d_6, 600 MHz)
Figure S20. COSY NMR spectrum of 4′-methylflavanone (**4**) (Acetone-d_6, 600 MHz)
Figure S21. COSY NMR spectrum of 4′-methylflavanone (**4**) (Acetone-d_6, 600 MHz)
Figure S22. HSQC NMR spectrum of 4′-methylflavanone (**4**) (Acetone-d_6, 151 MHz)
Figure S23. HSQC NMR spectrum of 4′-methylflavanone (**4**) (Acetone-d_6, 151 MHz)
Figure S24. HSQC NMR spectrum of 4′-methylflavanone (**4**) (Acetone-d_6, 151 MHz)
Figure S25. HMBC NMR spectrum of 4′-methylflavanone (**4**) (Acetone-d_6, 151 MHz)
Figure S26. HMBC NMR spectrum of 4′-methylflavanone (**4**) (Acetone-d_6, 151 MHz)
Figure S27. HMBC NMR spectrum of 4′-methylflavanone (**4**) (Acetone-d_6, 151 MHz)
Figure S28. ^{1}H NMR spectrum of 4′-methylflavone (**5**) (Acetone-d_6, 600 MHz)
Figure S29. ^{1}H NMR spectrum of 4′-methylflavone (**5**) (Acetone-d_6, 600 MHz)
Figure S30. ^{13}C NMR spectrum of 4′-methylflavone (**5**) (Acetone-d_6, 151 MHz)
Figure S31. ^{13}C NMR spectrum of 4′-methylflavone (**5**) (Acetone-d_6, 151 MHz)
Figure S32. COSY NMR spectrum of 4′-methylflavone (**5**) (Acetone-d_6, 600 MHz)
Figure S33. COSY NMR spectrum of 4′-methylflavone (**5**) (Acetone-d_6, 600 MHz)
Figure S34. COSY NMR spectrum of 4′-methylflavone (**5**) (Acetone-d_6, 600 MHz)
Figure S35. HSQC NMR spectrum of 4′-methylflavone (**5**) (Acetone-d_6, 151 MHz)
Figure S36. HSQC NMR spectrum of 4′-methylflavone (**5**) (Acetone-d_6, 151 MHz)

Figure S37. HSQC NMR spectrum of 4′-methylflavone (**5**) (Acetone-d_6, 151 MHz)
Figure S38. HMBC NMR spectrum of 4′-methylflavone (**5**) (Acetone-d_6, 151 MHz)
Figure S39. HMBC NMR spectrum of 4′-methylflavanone (**5**) (Acetone-d_6, 151 MHz)
Figure S40. HMBC NMR spectrum of 4′-methylflavanone (**5**) (Acetone-d_6, 151 MHz)
Figure S41. The fluorescence intensity of 4′-methylflavanone, λ_{ex}=360 nm
Table S1. Selected bands of ATR-FTIR spectra of compounds/RBCMs system and RBCMs, concentration of compounds 25 μM

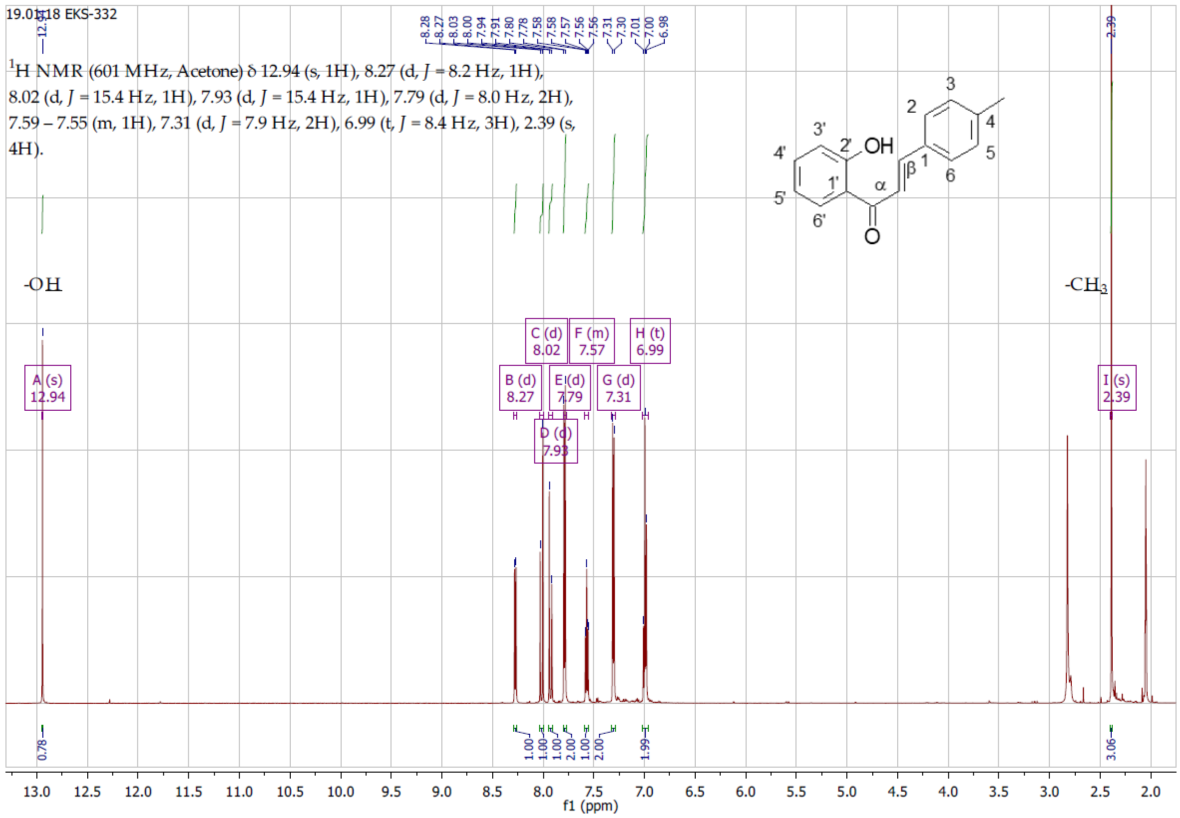

Figure S1. ¹H NMR spectrum of 2′-hydroxy-4-methylchalcone (**3**) (Acetone-d_6, 600 MHz)

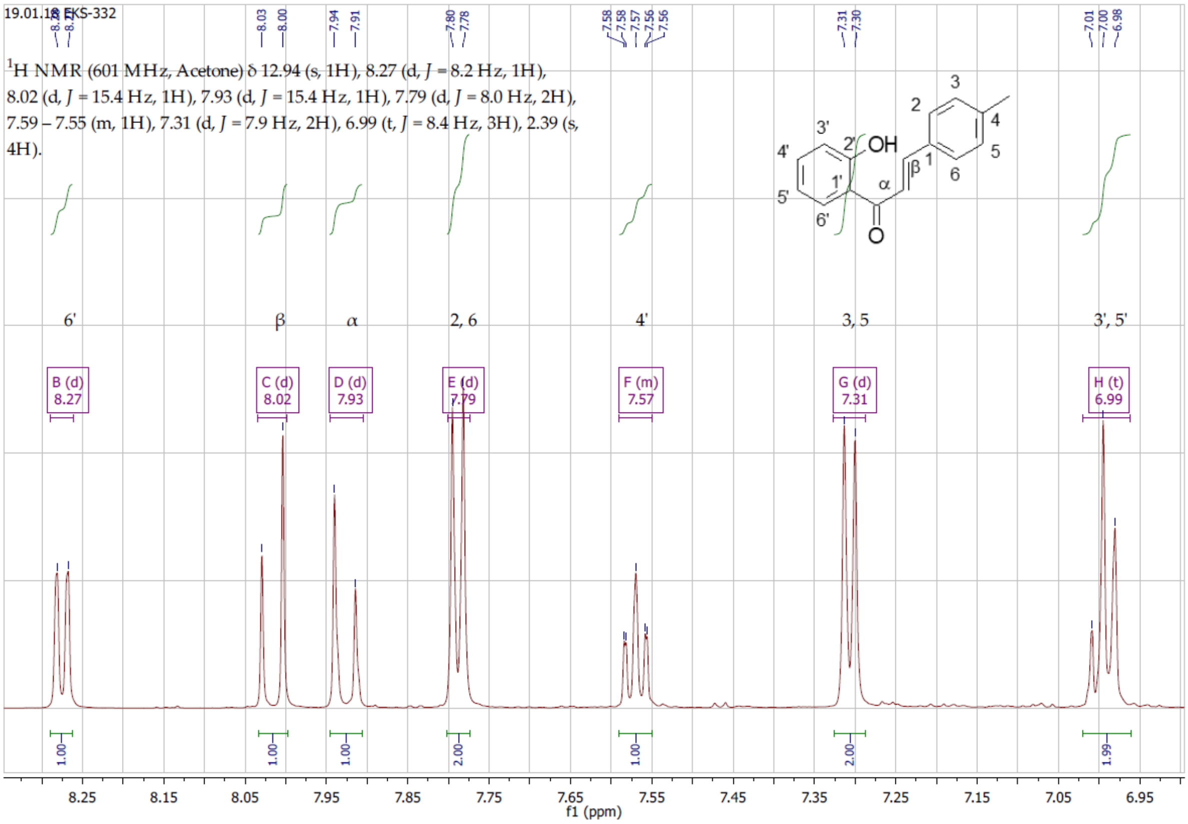

Figure S2. ¹H NMR spectrum of 2′-hydroxy-4-methylchalcone (**3**) (Acetone-d_6, 600 MHz)

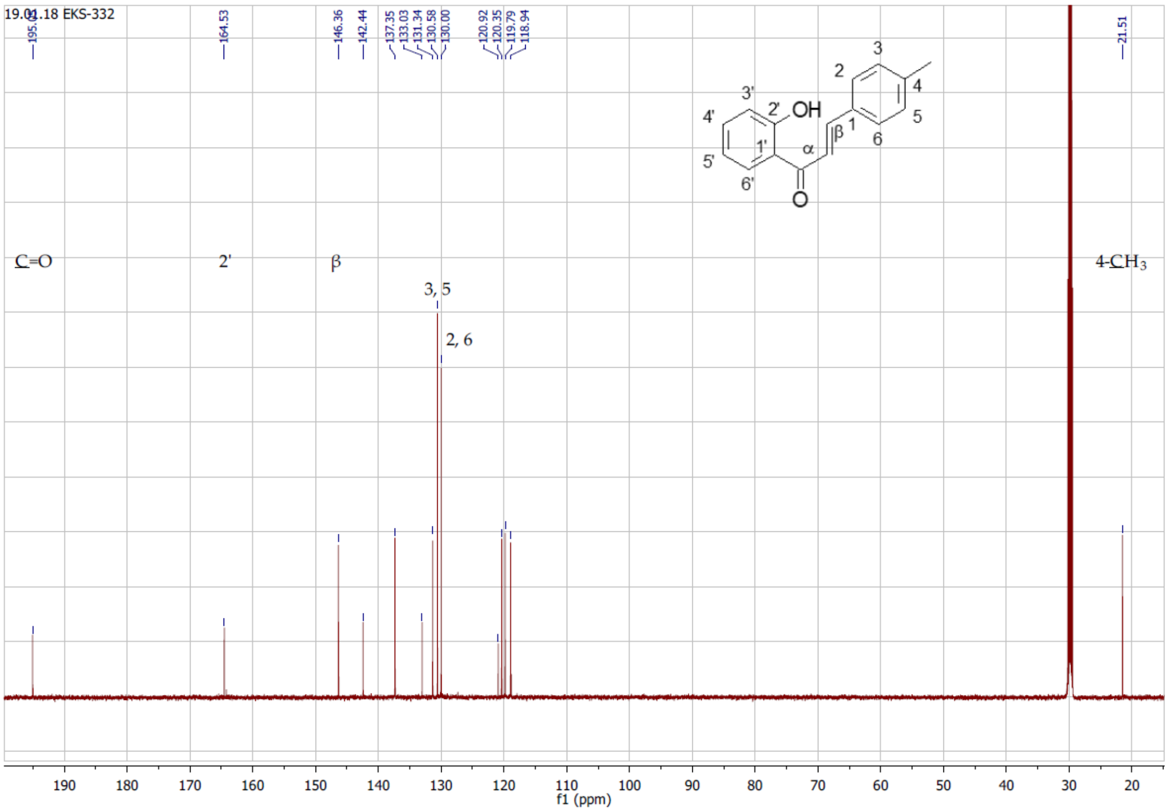

Figure S3. ^{13}C NMR spectrum of 2′-hydroxy-4-methylchalcone (**3**) (Acetone-d$_6$, 151 MHz)

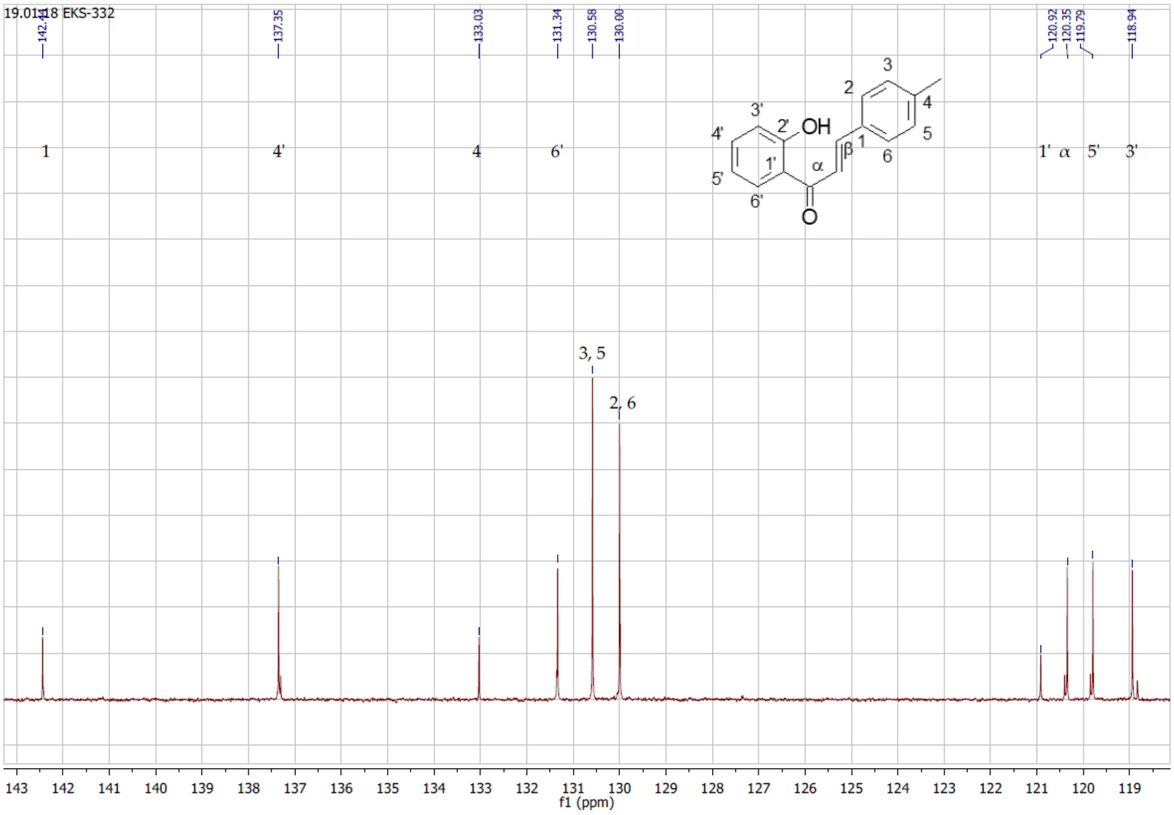

Figure S4. ^{13}C NMR spectrum of 2′-hydroxy-4-methylchalcone (**3**) (Acetone-d$_6$, 151 MHz)

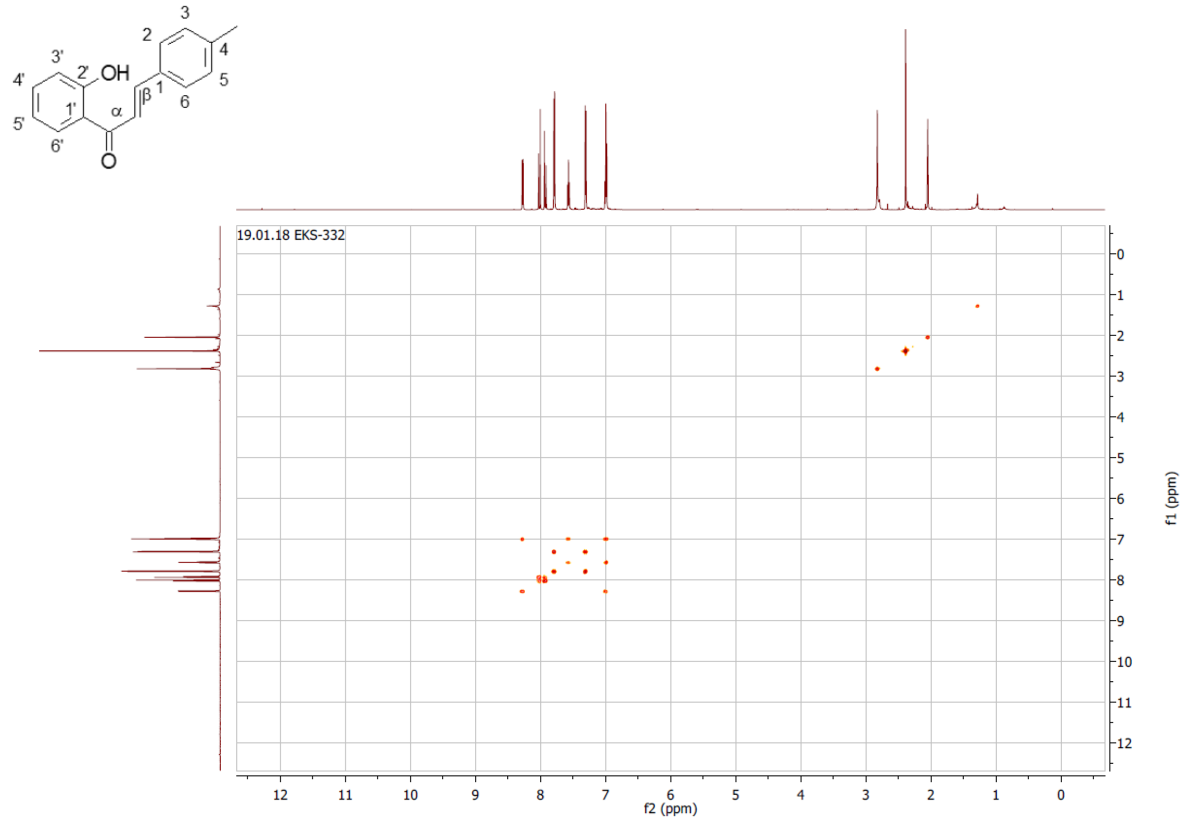

Figure S5. COSY NMR spectrum of 2′-hydroxy-4-methylchalcone (**3**) (Acetone-d$_6$, 600 MHz)

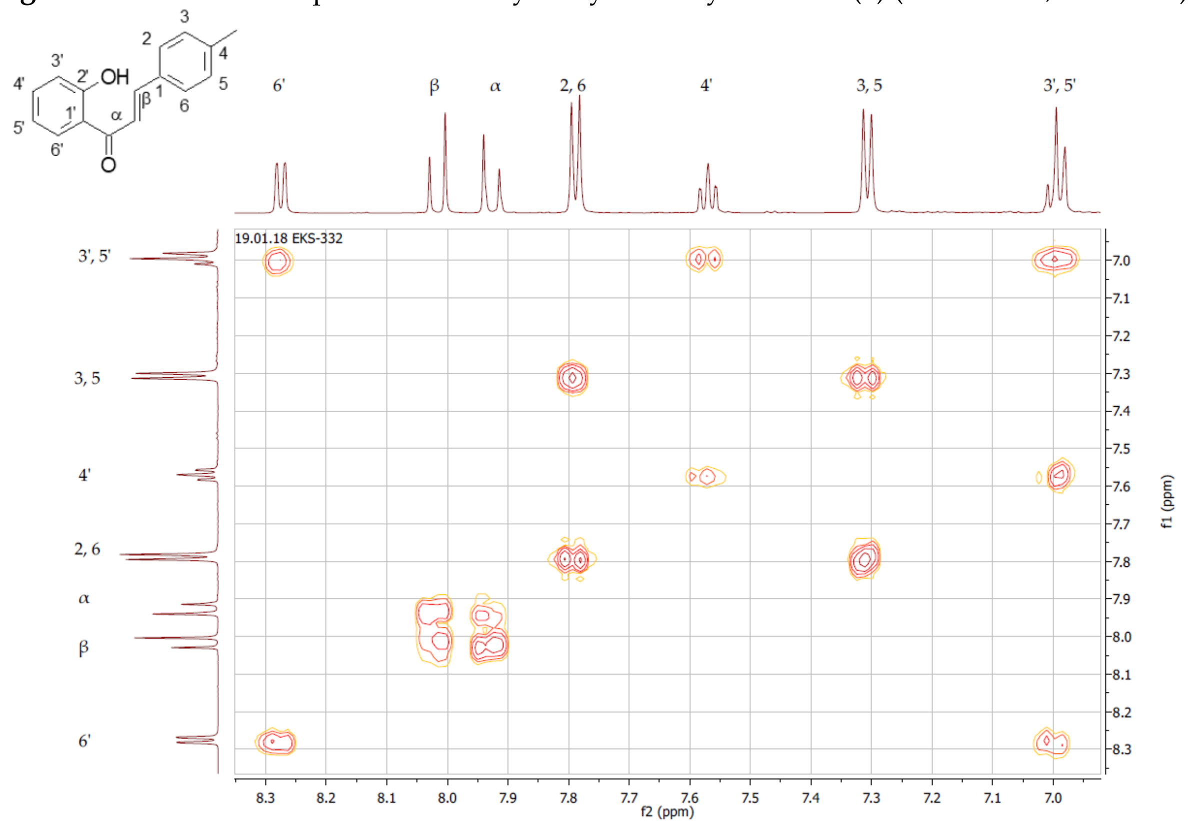

Figure S6. COSY NMR spectrum of 2′-hydroxy-4-methylchalcone (**3**) (Acetone-d$_6$, 600 MHz)

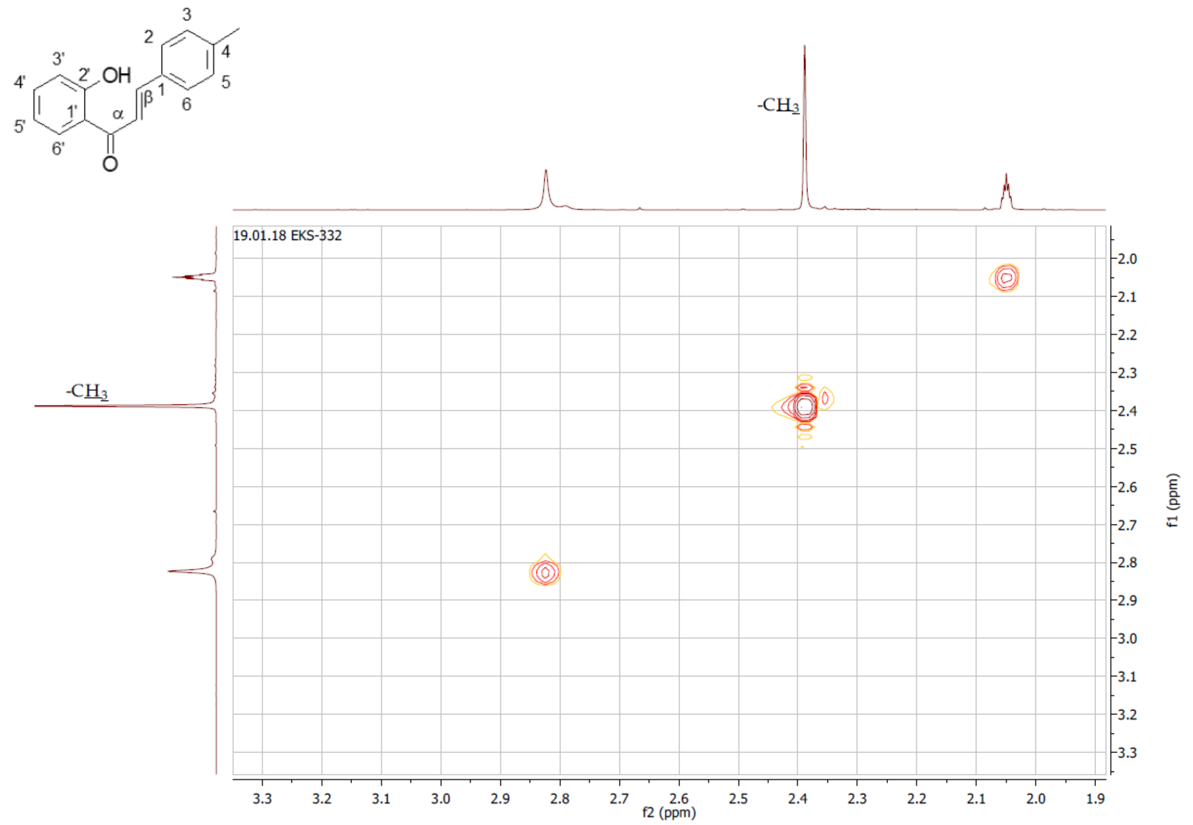

Figure S7. COSY NMR spectrum of 2′-hydroxy-4-methylchalcone (**3**) (Acetone-d6, 600 MHz)

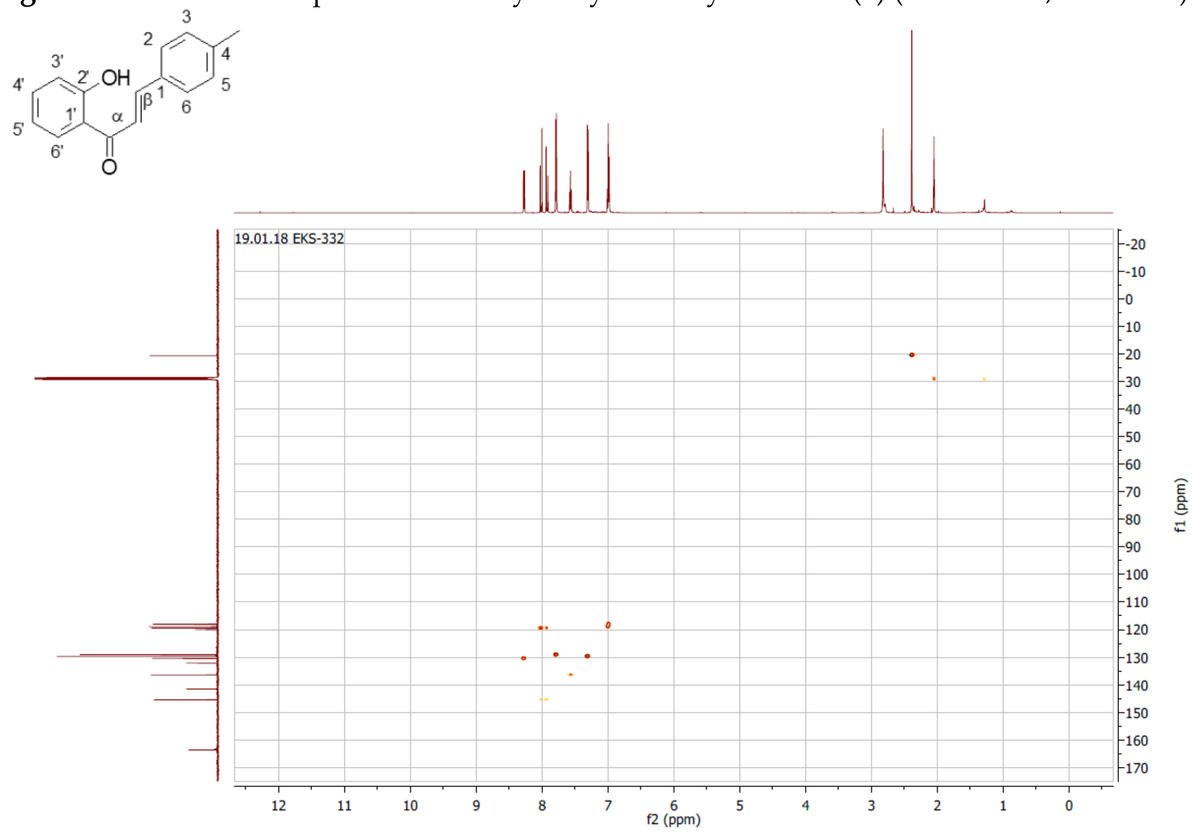

Figure S8. HSQC NMR spectrum of 2′-hydroxy-4-methylchalcone (**3**) (Acetone-d6, 151 MHz)

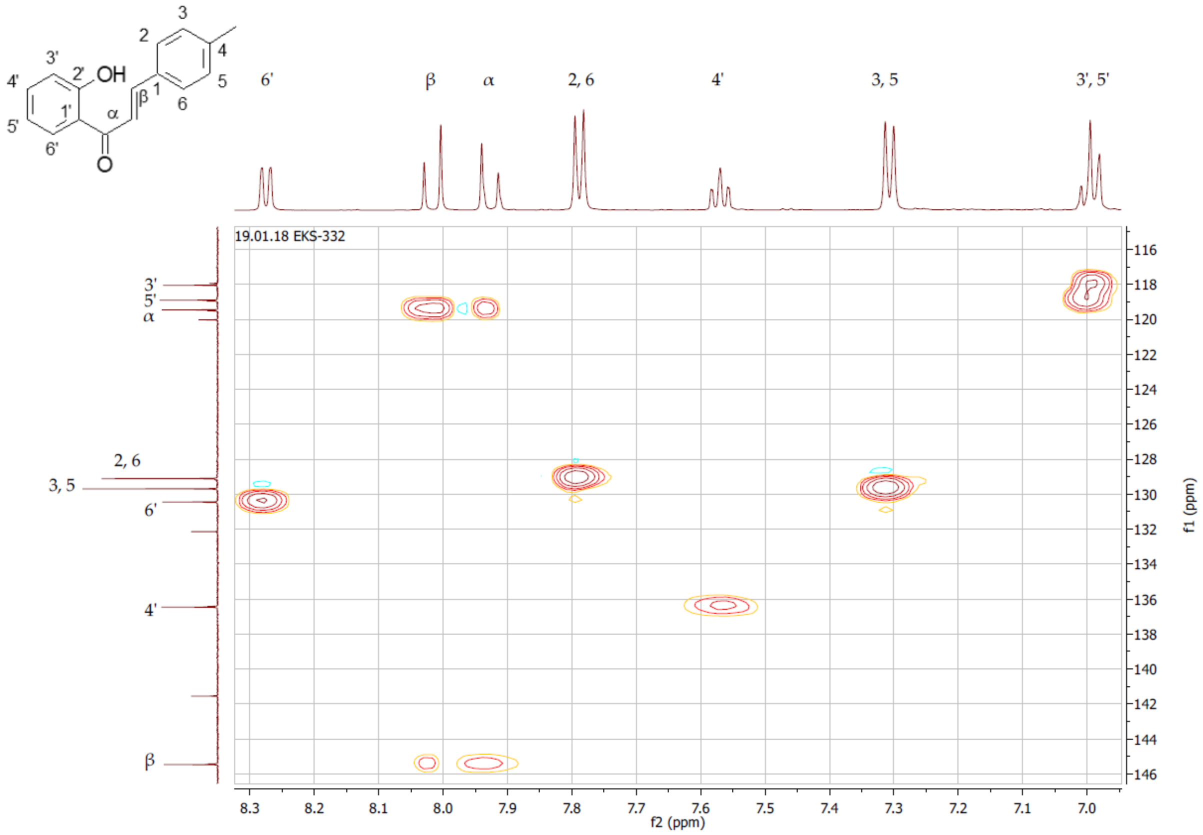

Figure S9. HSQC NMR spectrum of 2′-hydroxy-4-methylchalcone (**3**) (Acetone-d$_6$, 151 MHz)

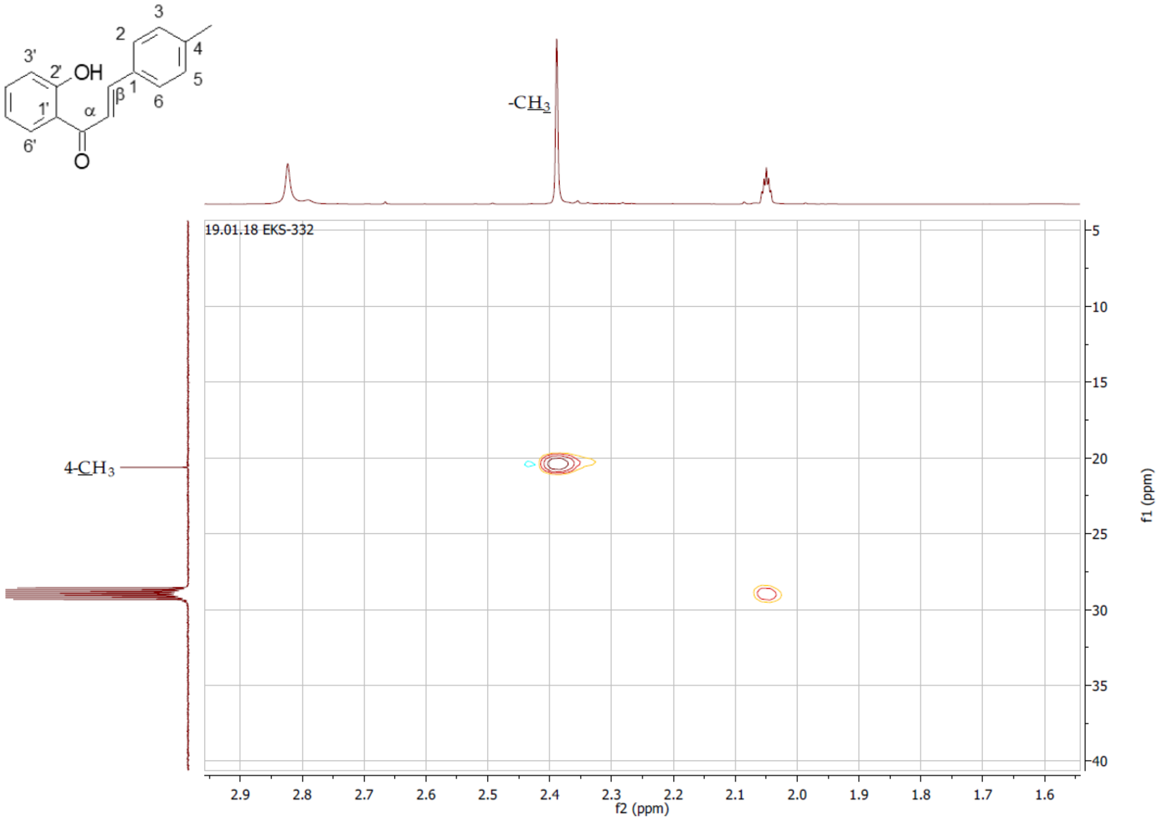

Figure S10. HSQC NMR spectrum of 2′-hydroxy-4-methylchalcone (**3**) (Acetone-d$_6$, 151 MHz)

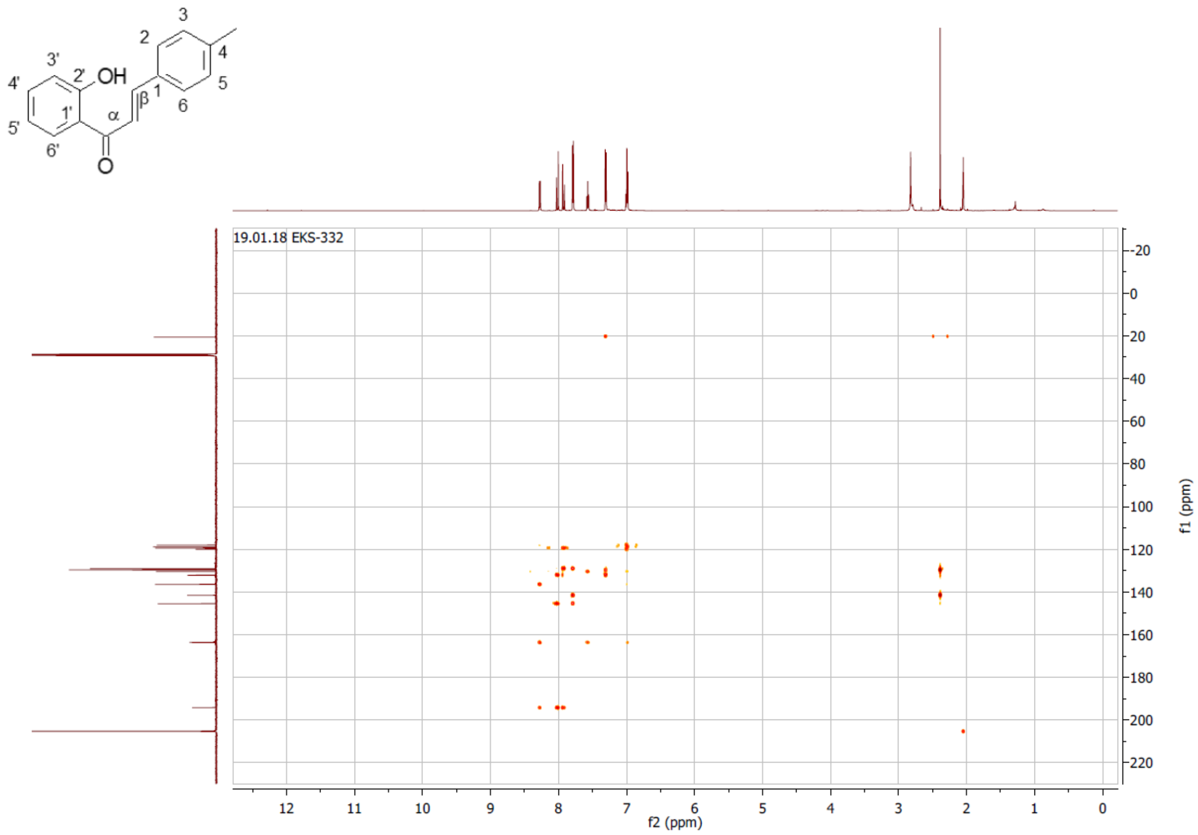

Figure S11. HMBC NMR spectrum of 2′-hydroxy-4-methylchalcone (**3**) (Acetone-d₆, 151 MHz)

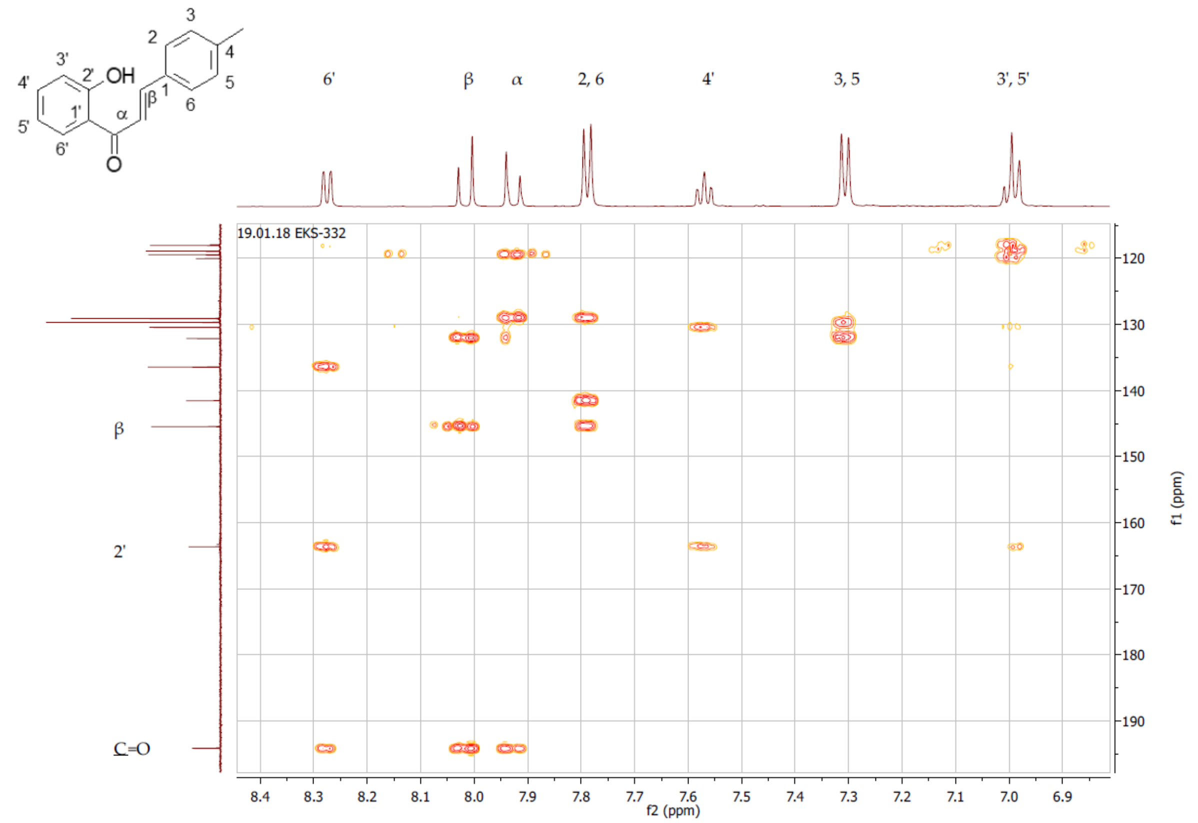

Figure S12. HMBC NMR spectrum of 2′-hydroxy-4-methylchalcone (**3**) (Acetone-d₆, 151 MHz)

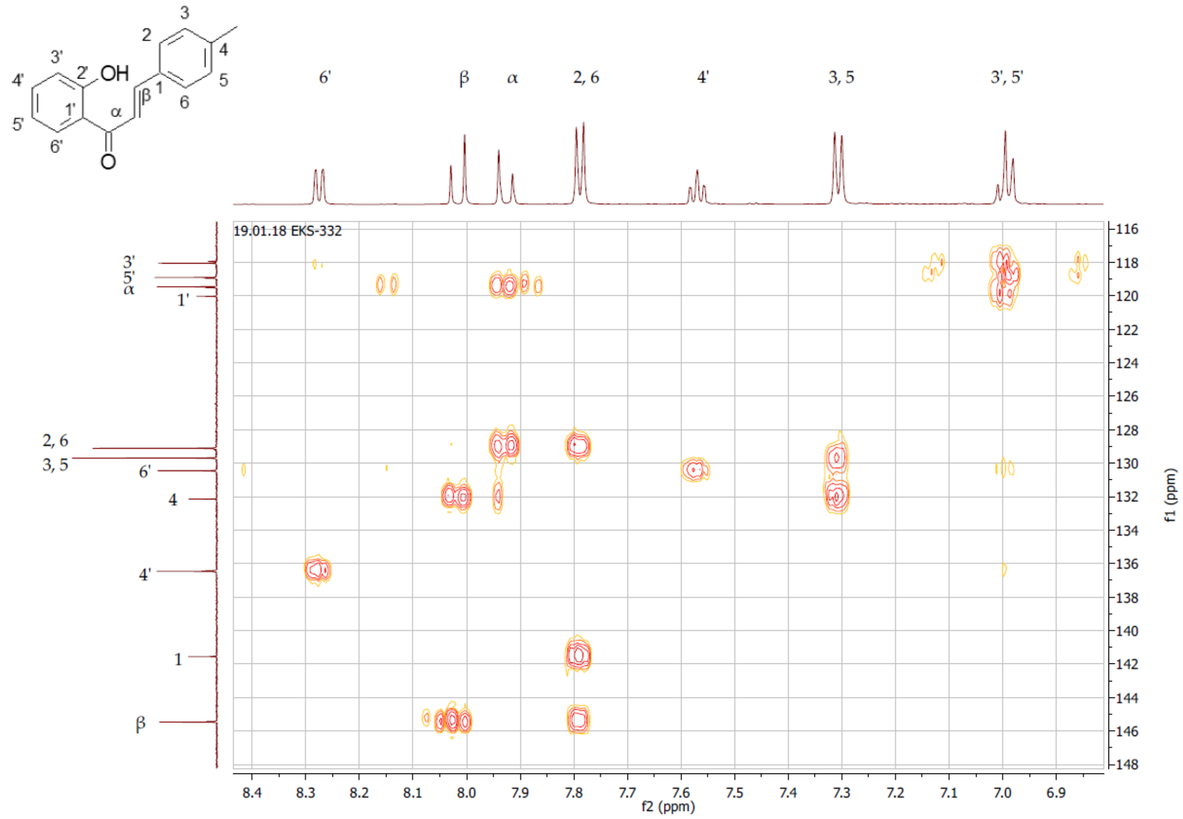

Figure S13. HMBC NMR spectrum of 2′-hydroxy-4-methylchalcone (**3**) (Acetone-d$_6$, 151 MHz)

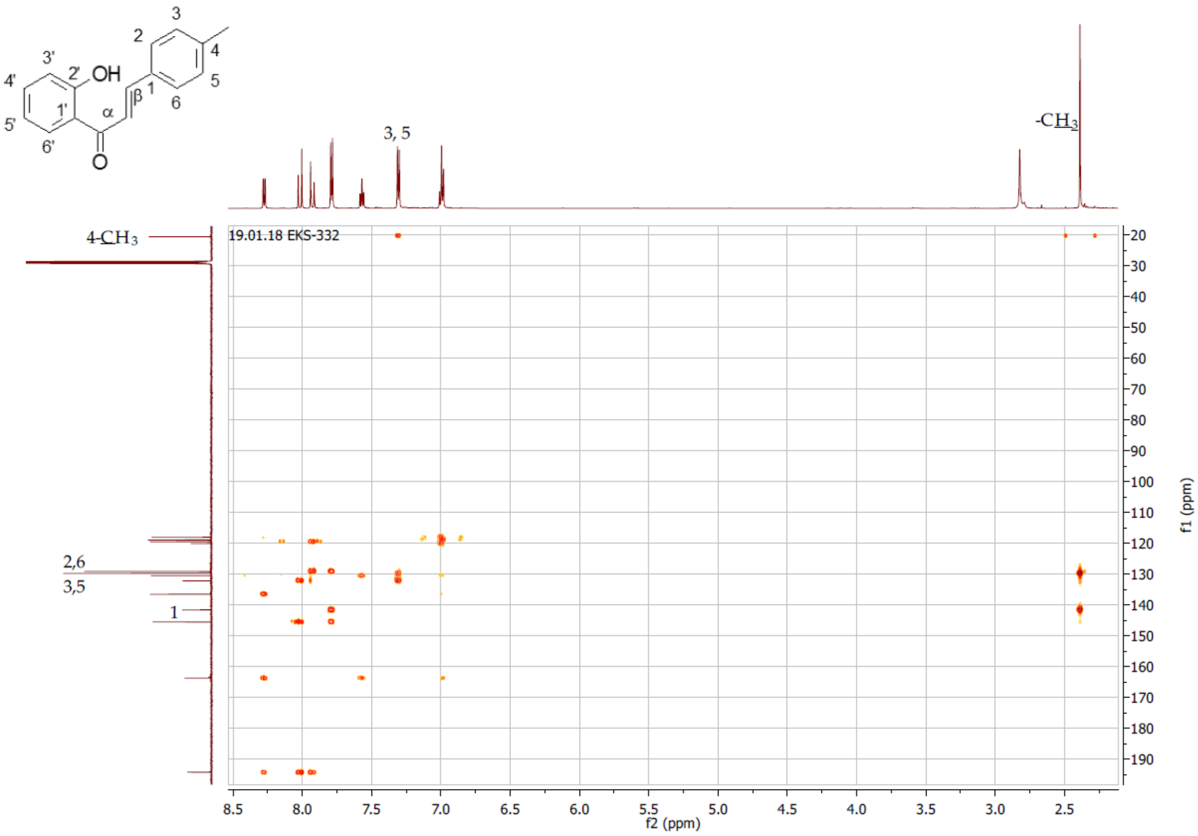

Figure S14. HMBC NMR spectrum of 2′-hydroxy-4-methylchalcone (**3**) (Acetone-d$_6$, 151 MHz)

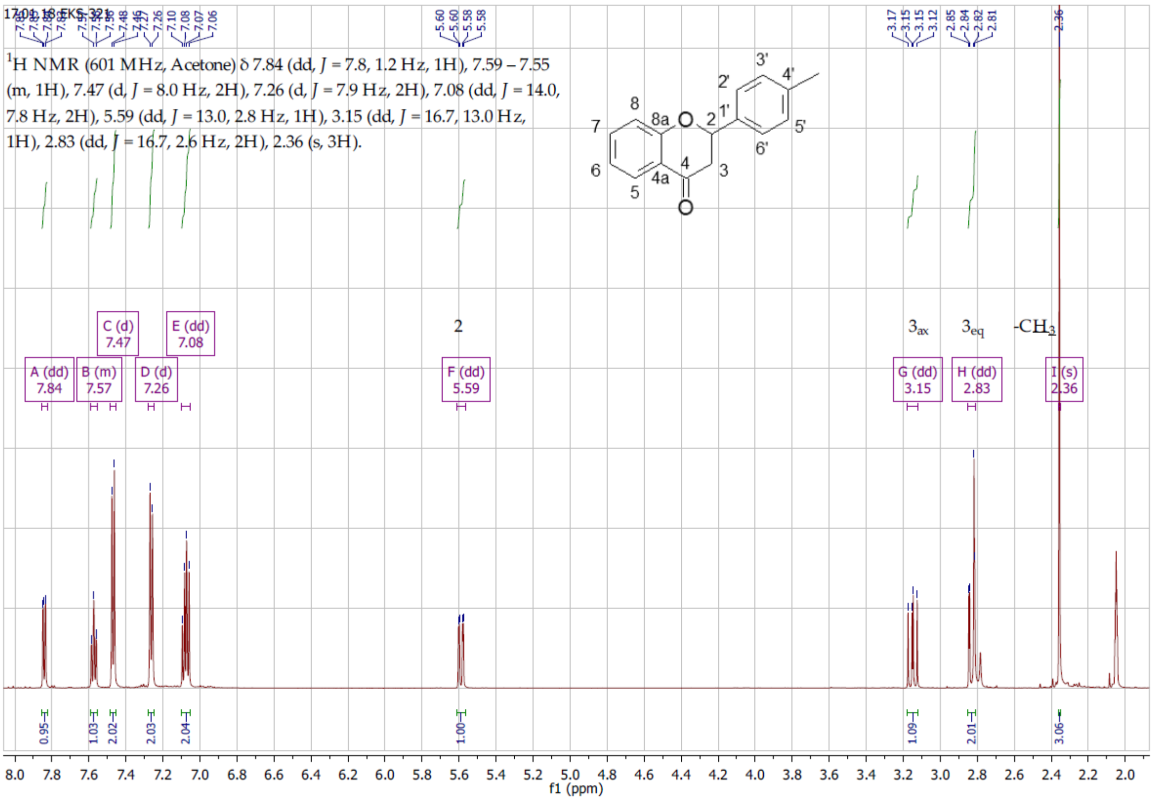

Figure S15. ^{1}H NMR spectrum of 4′-methylflavanone (**4**) (Acetone-d$_6$, 600 MHz)

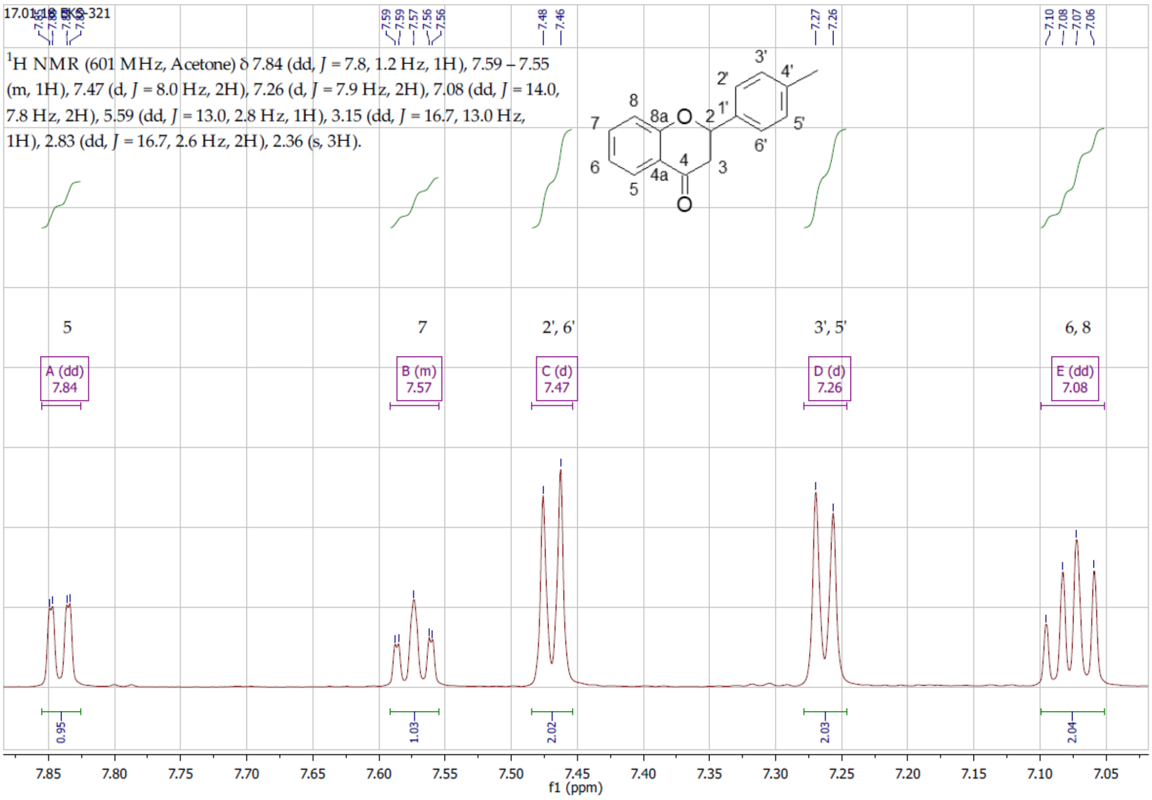

Figure S16. ^{1}H NMR spectrum of 4′-methylflavanone (**4**) (Acetone-d$_6$, 600 MHz)

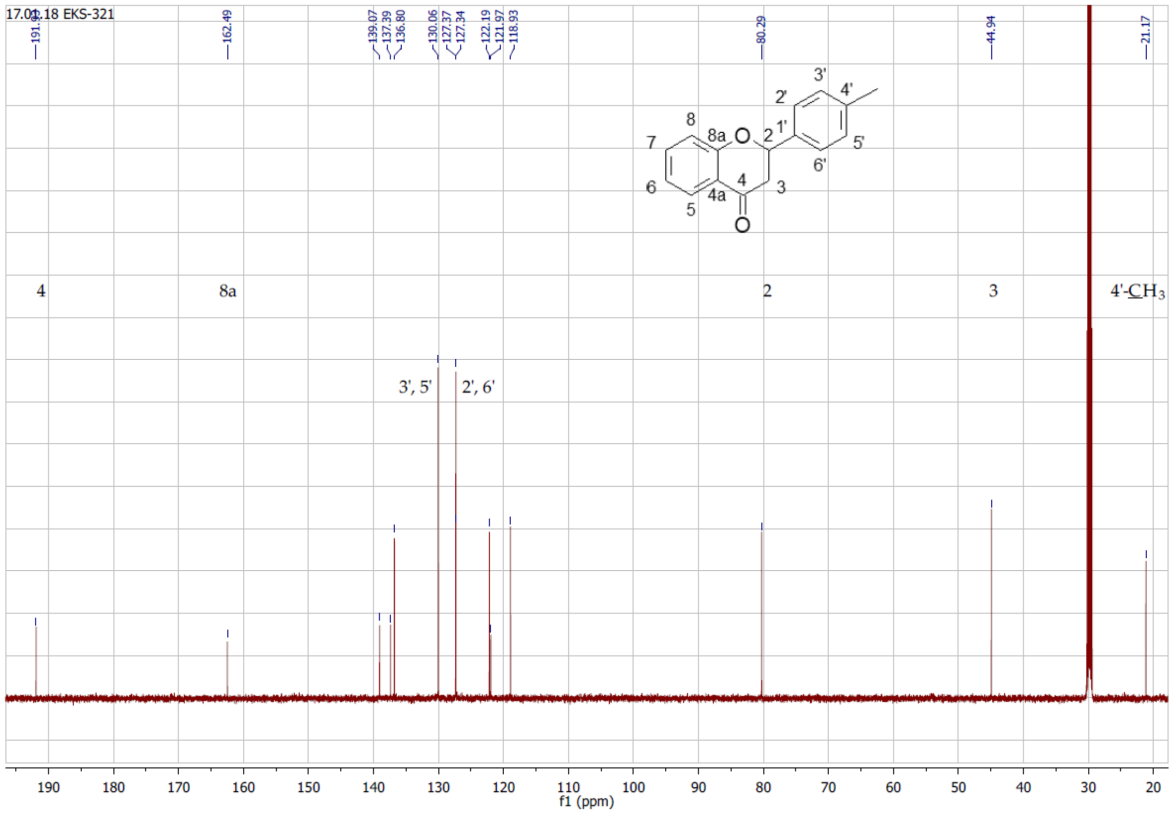

Figure S17. ^{13}C NMR spectrum of 4′-methylflavanone (**4**) (Acetone-d$_6$, 151 MHz)

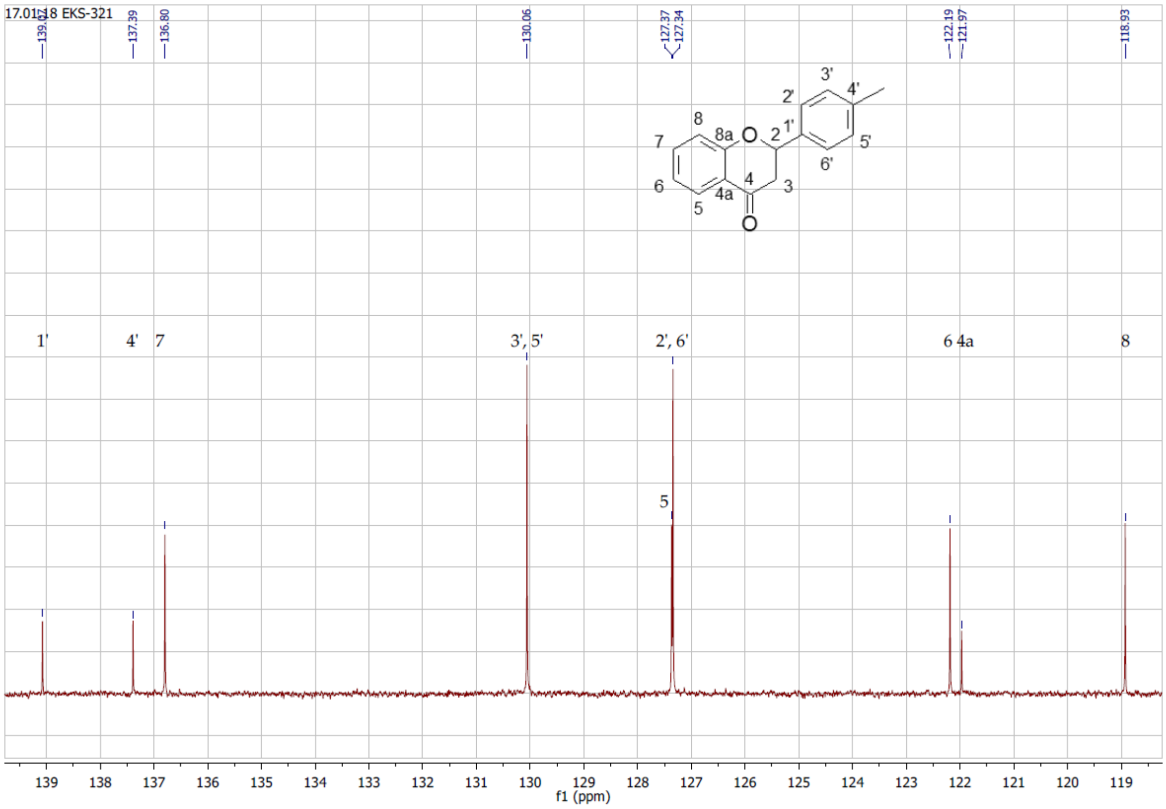

Figure S18. ^{13}C NMR spectrum of 4′-methylflavanone (**4**) (Acetone-d$_6$, 151 MHz)

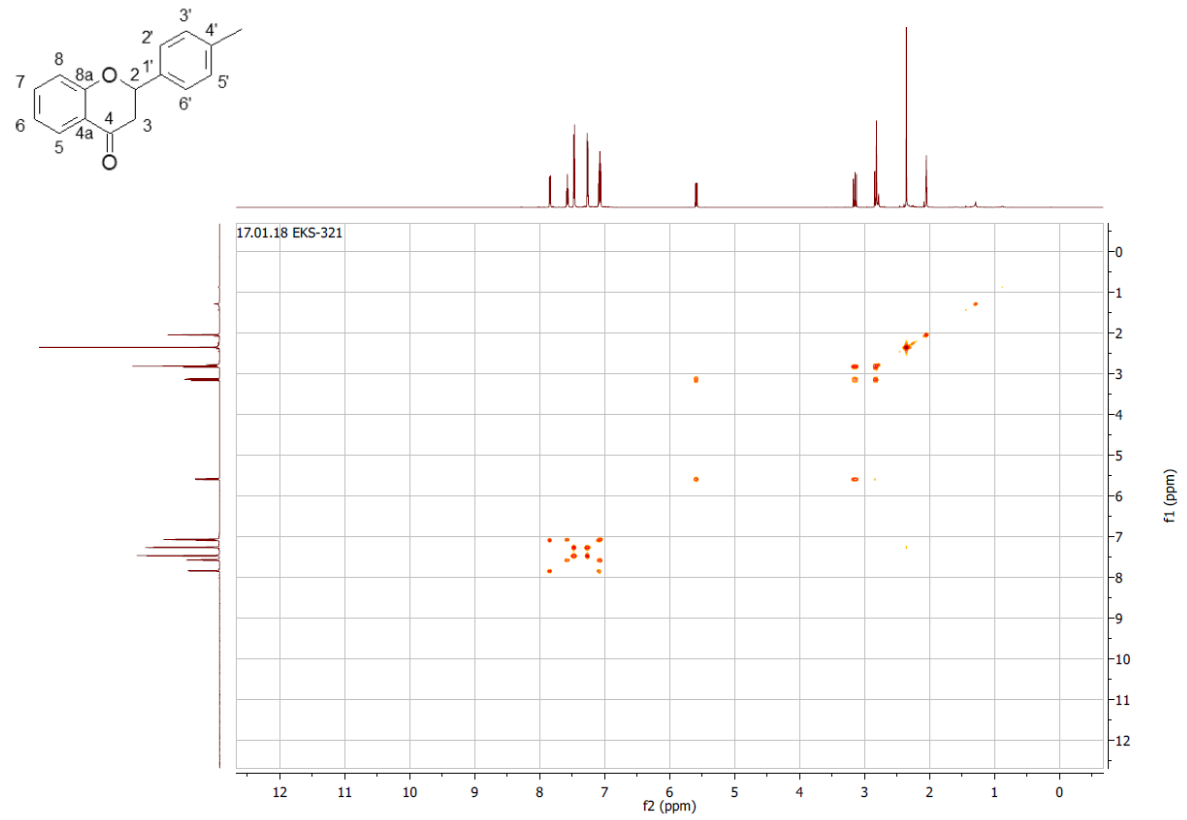

Figure S19. COSY NMR spectrum of 4′-methylflavanone (**4**) (Acetone-d_6, 600 MHz)

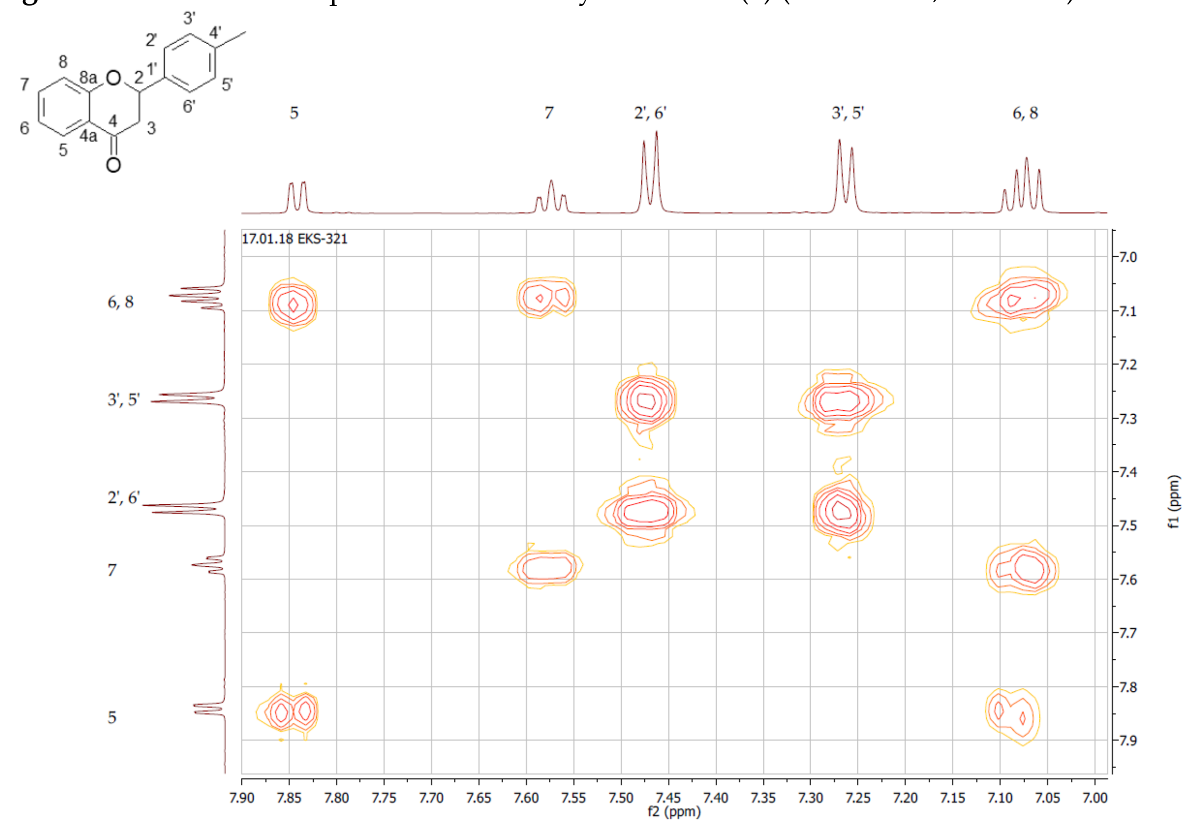

Figure S20. COSY NMR spectrum of 4′-methylflavanone (**4**) (Acetone-d_6, 600 MHz)

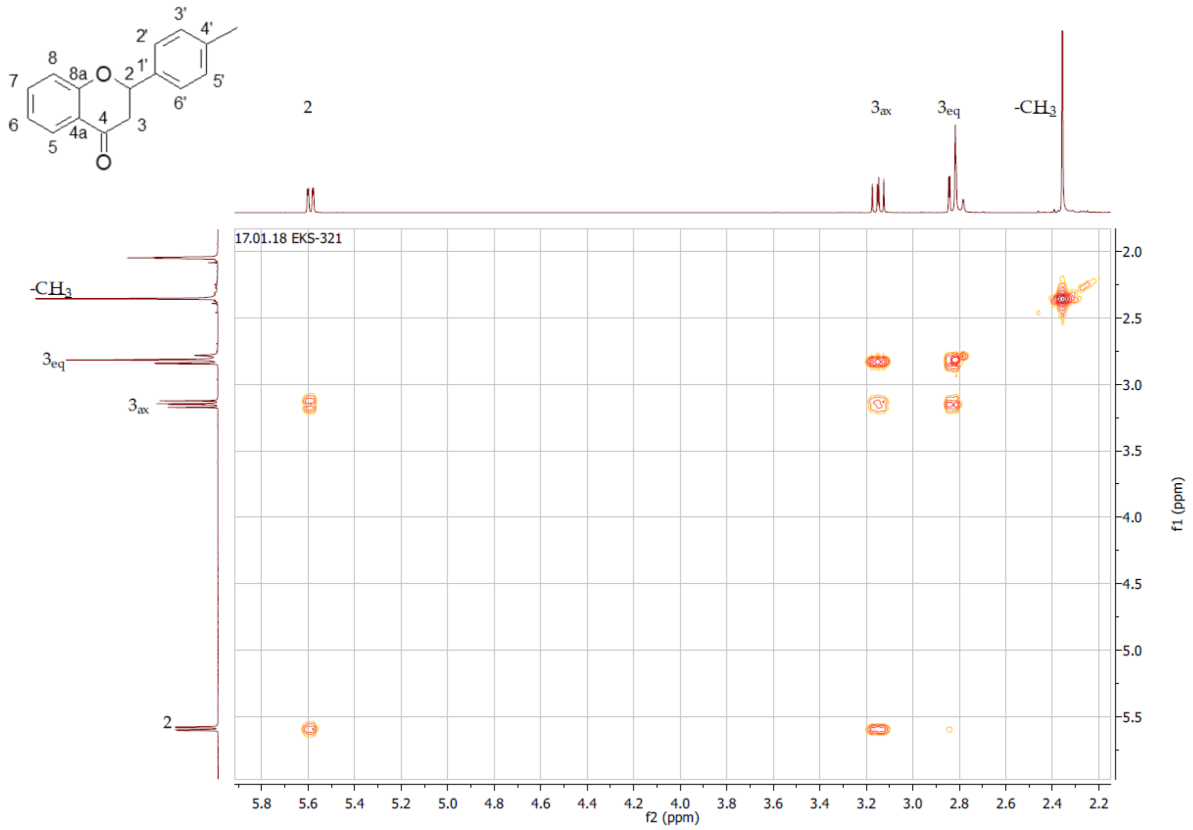

Figure S21. COSY NMR spectrum of 4′-methylflavanone (**4**) (Acetone-d6, 600 MHz)

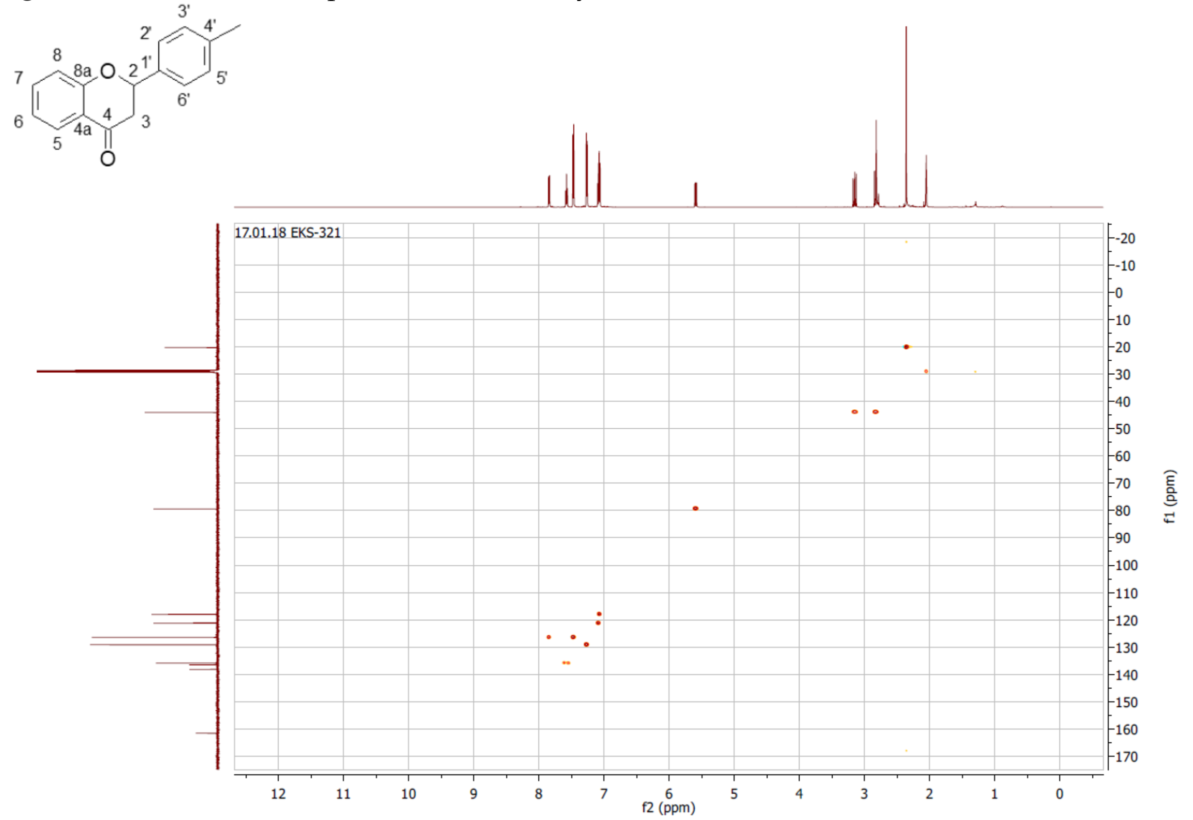

Figure S22. HSQC NMR spectrum of 4′-methylflavanone (**4**) (Acetone-d6, 151 MHz)

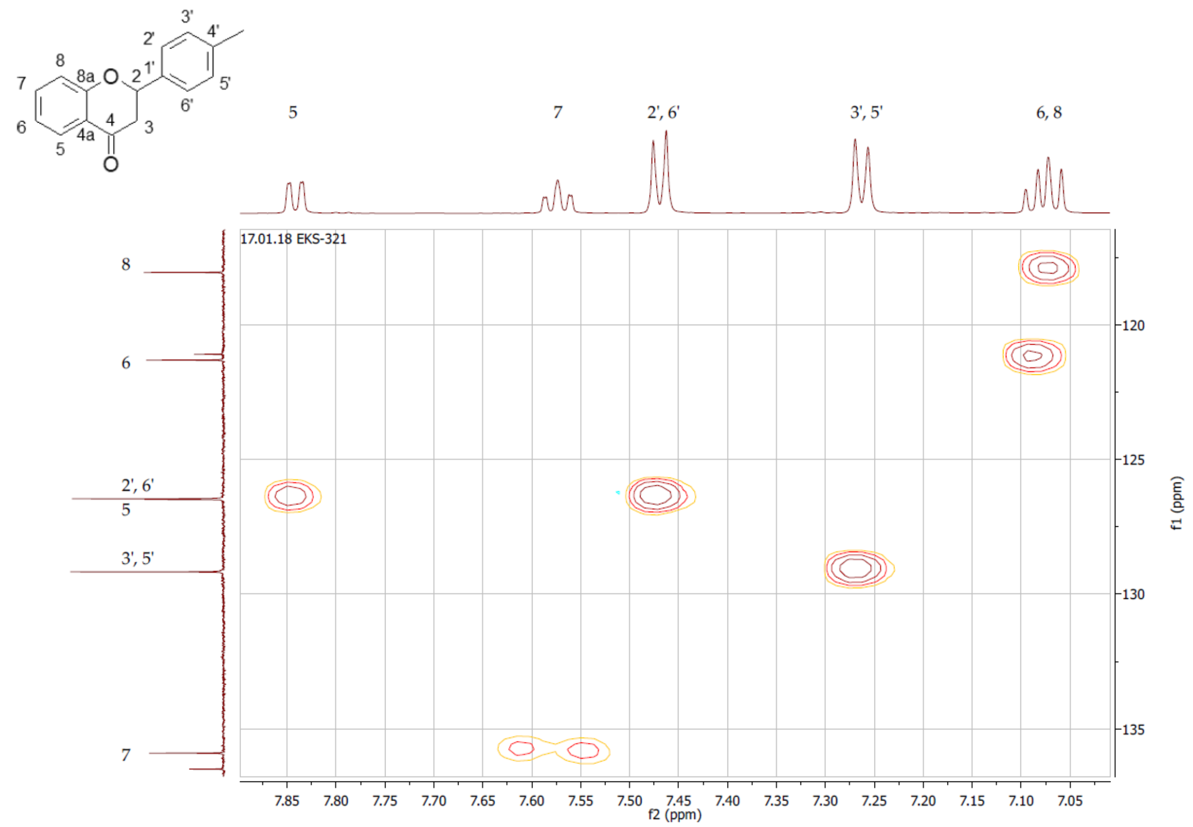

Figure S23. HSQC NMR spectrum of 4′-methylflavanone (**4**) (Acetone-d6, 151 MHz)

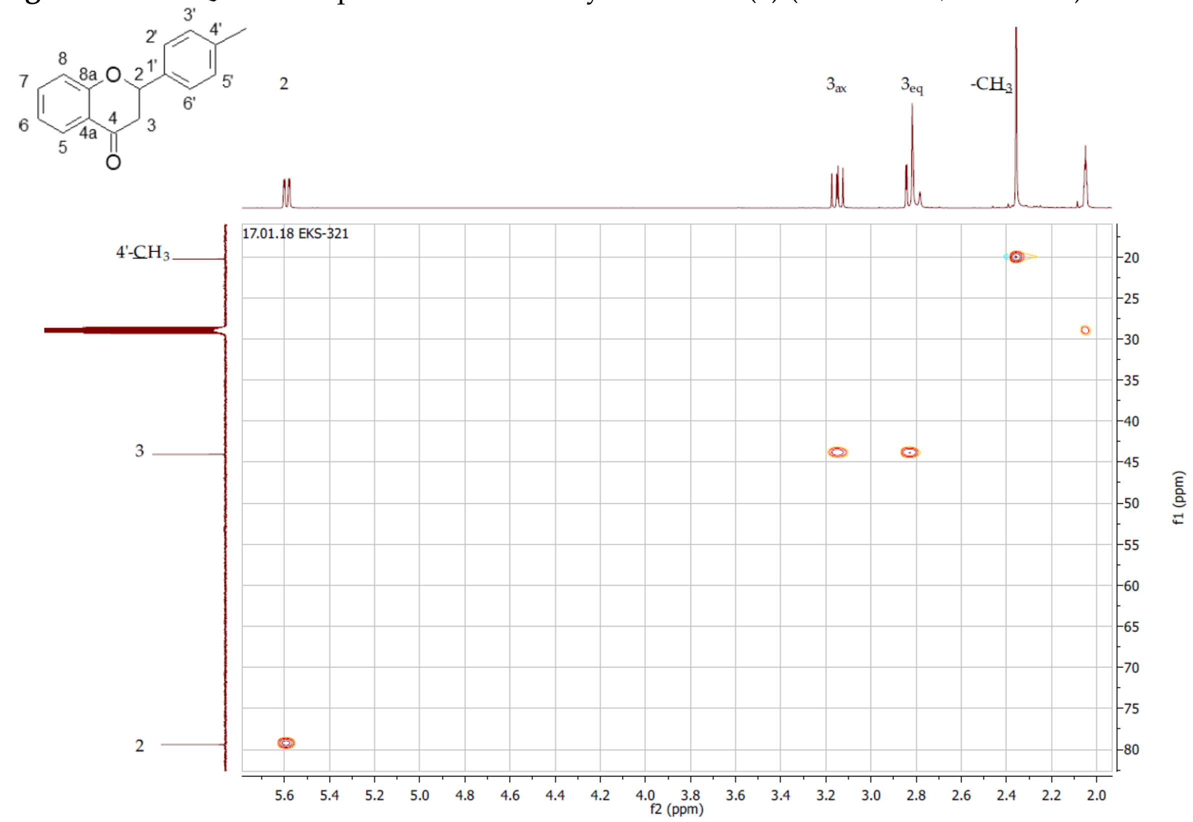

Figure S24. HSQC NMR spectrum of 4′-methylflavanone (**4**) (Acetone-d6, 151 MHz)

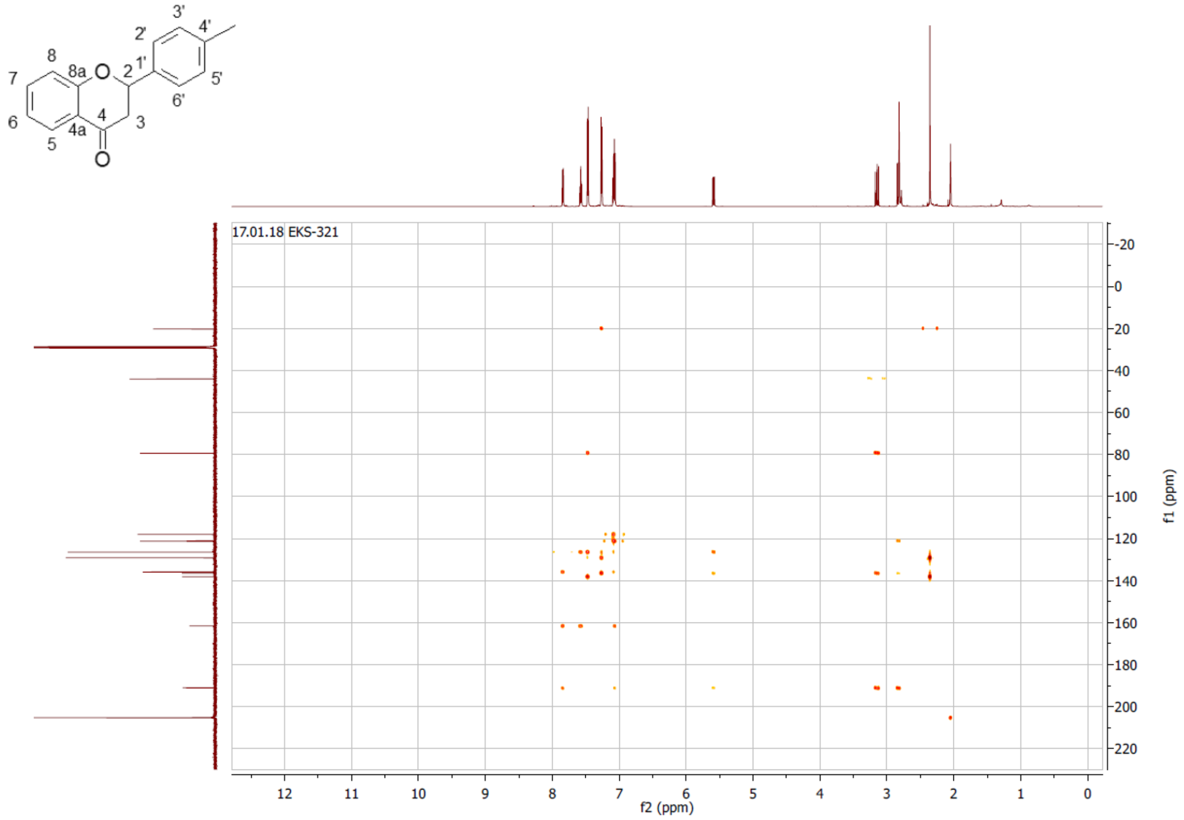

Figure S25. HMBC NMR spectrum of 4′-methylflavanone (**4**) (Acetone-d6, 151 MHz)

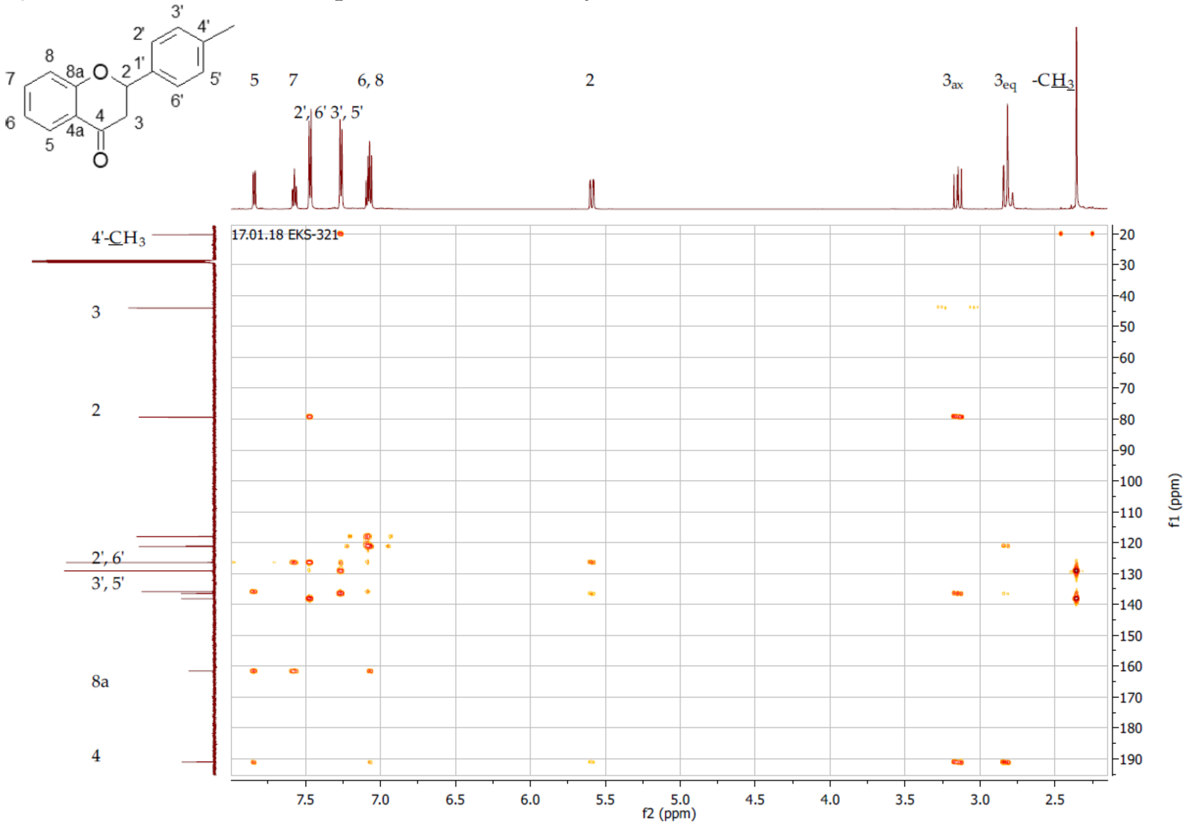

Figure S26. HMBC NMR spectrum of 4′-methylflavanone (**4**) (Acetone-d6, 151 MHz)

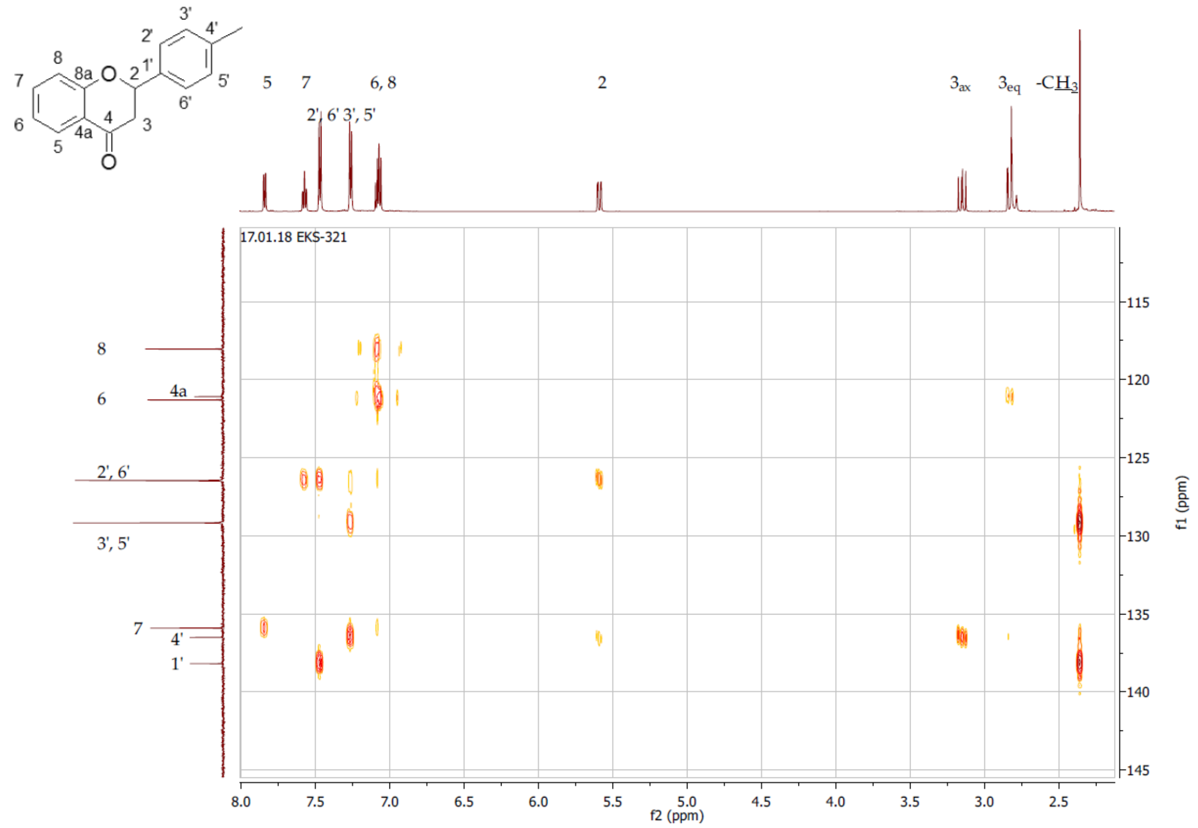

Figure S27. HMBC NMR spectrum of 4′-methylflavanone (**4**) (Acetone-d₆, 151 MHz)

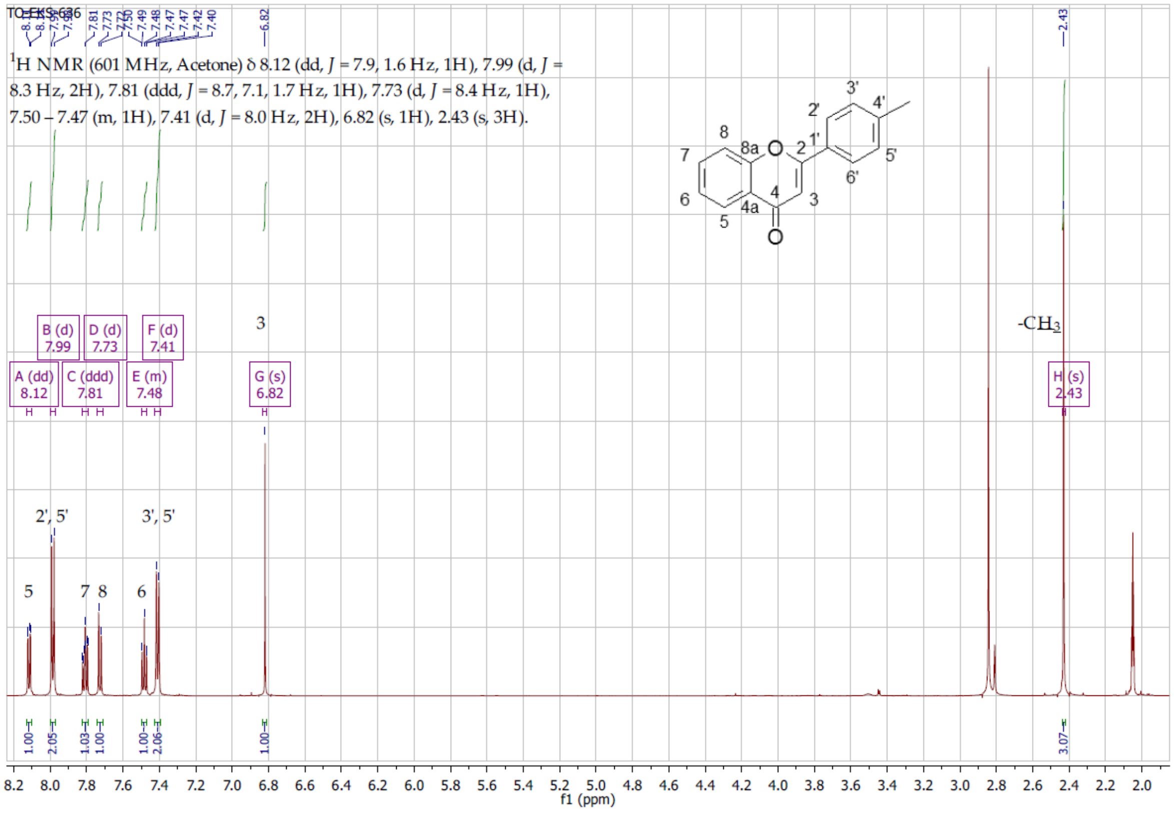

Figure S28. ¹H NMR spectrum of 4′-methylflavone (**5**) (Acetone-d₆, 600 MHz)

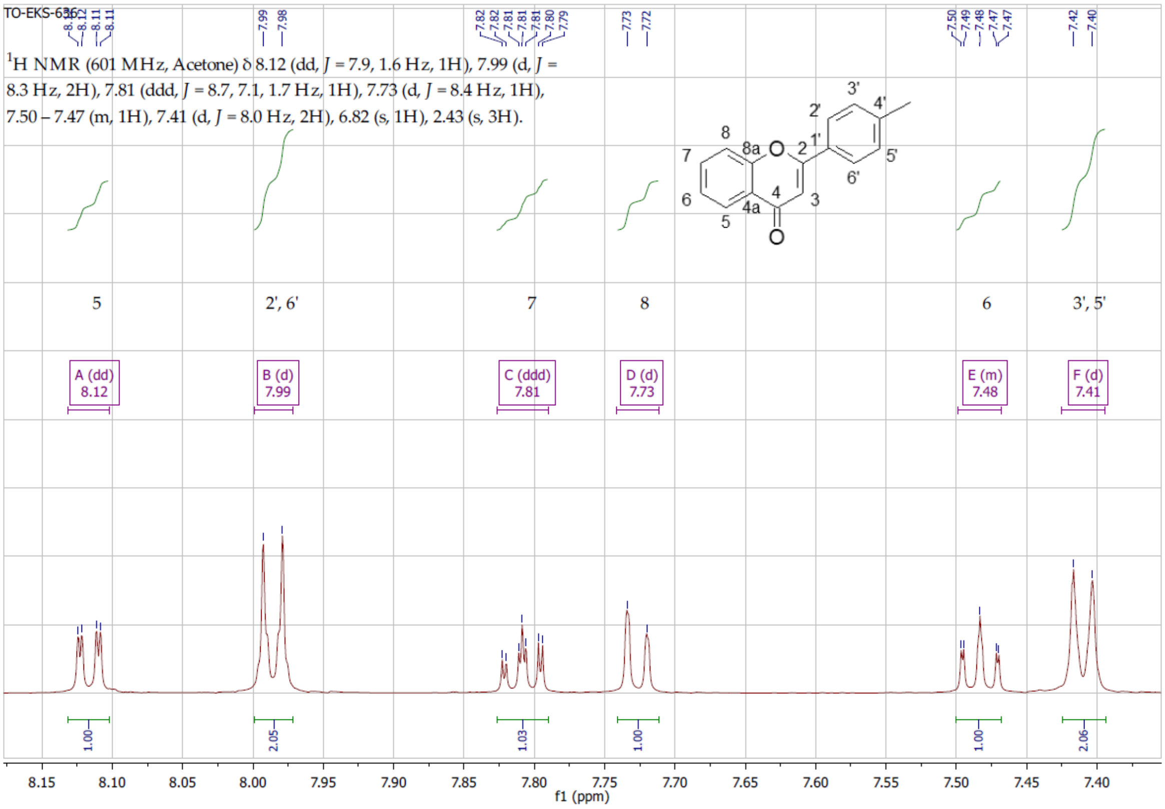

Figure S29. ^{1}H NMR spectrum of 4′-methylflavone (**5**) (Acetone-d$_6$, 600 MHz)

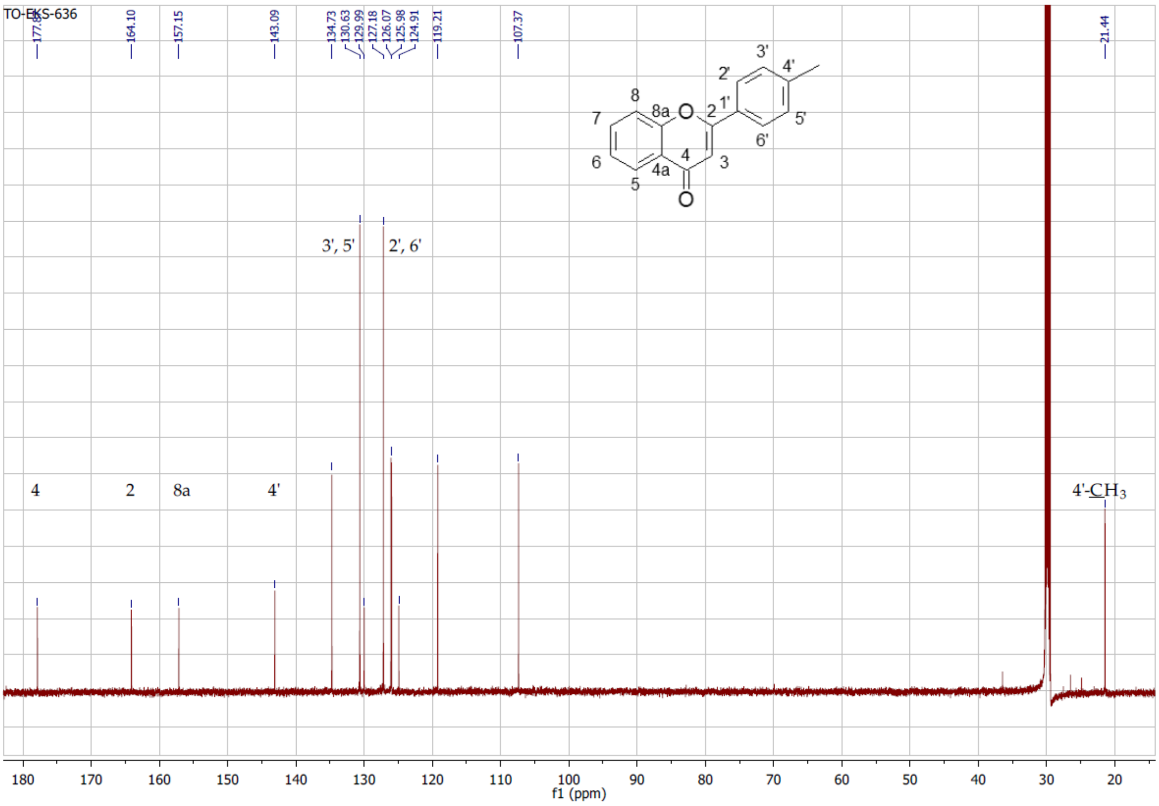

Figure S30. ^{13}C NMR spectrum of 4′-methylflavone (**5**) (Acetone-d$_6$, 151 MHz)

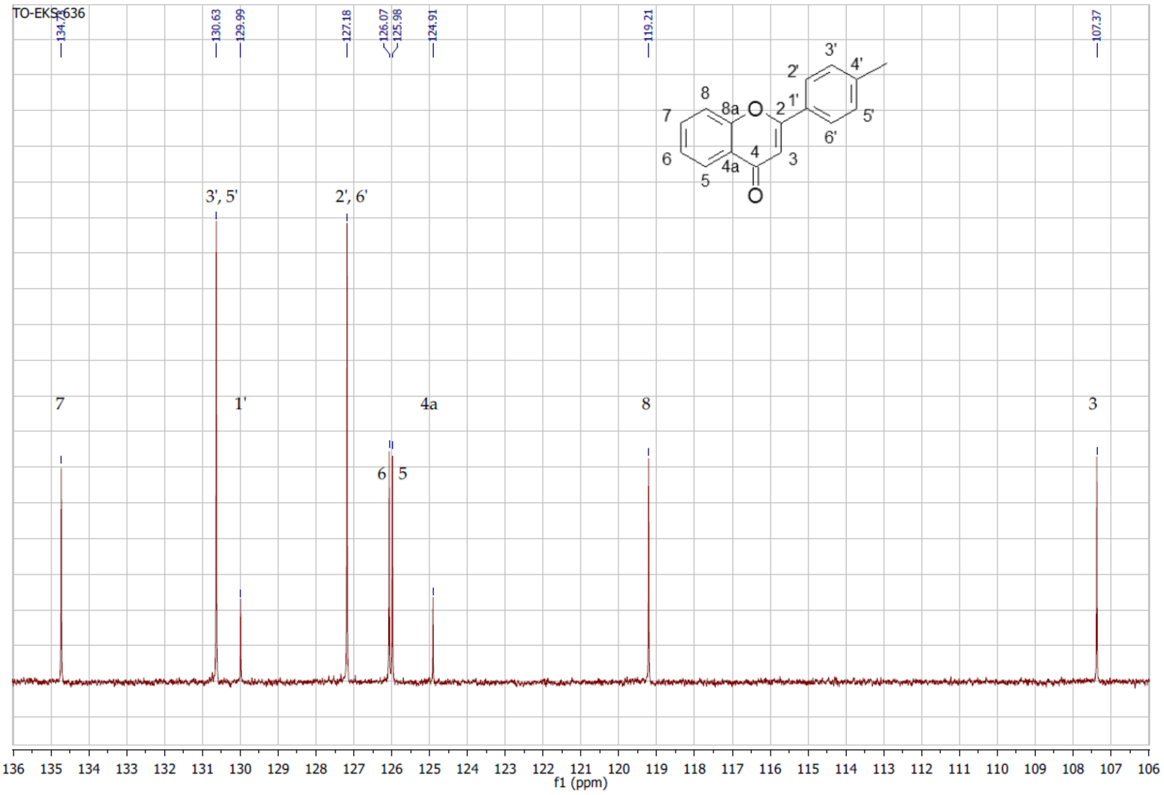

Figure S31. ^{13}C NMR spectrum of 4′-methylflavone (**5**) (Acetone-d_6, 151 MHz)

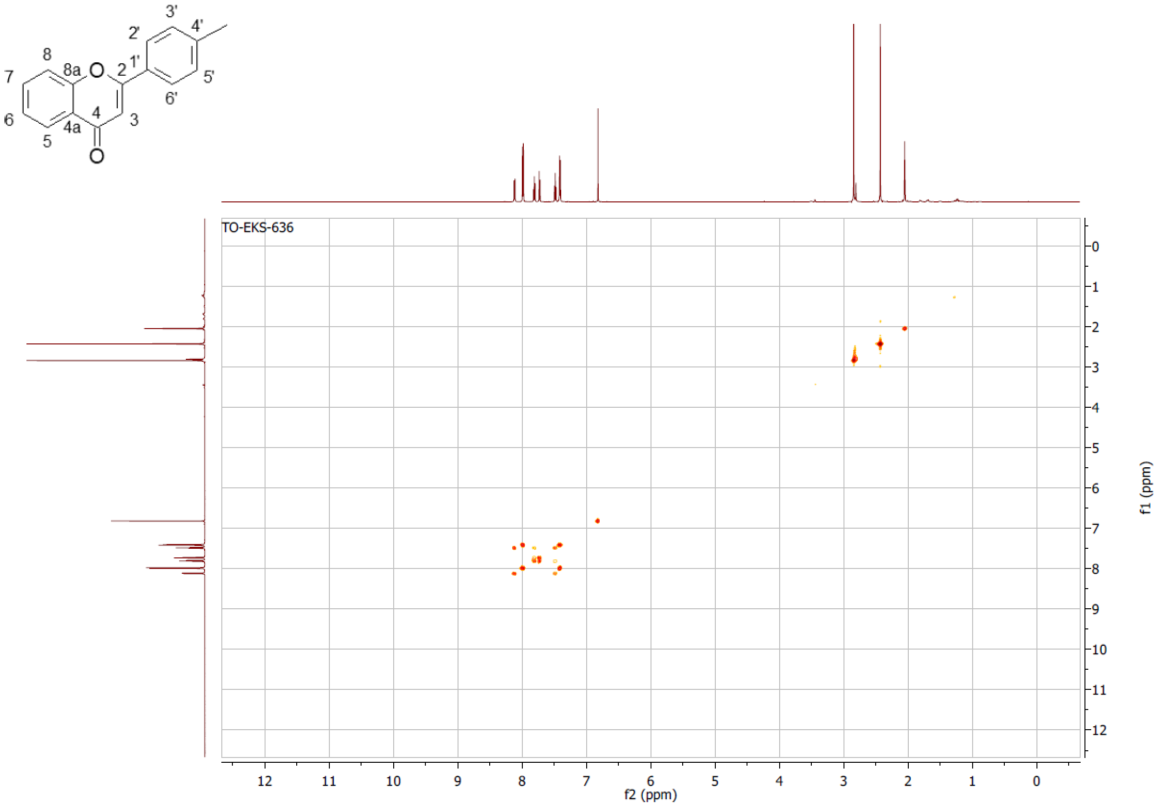

Figure S32. COSY NMR spectrum of 4′-methylflavone (**5**) (Acetone-d_6, 600 MHz)

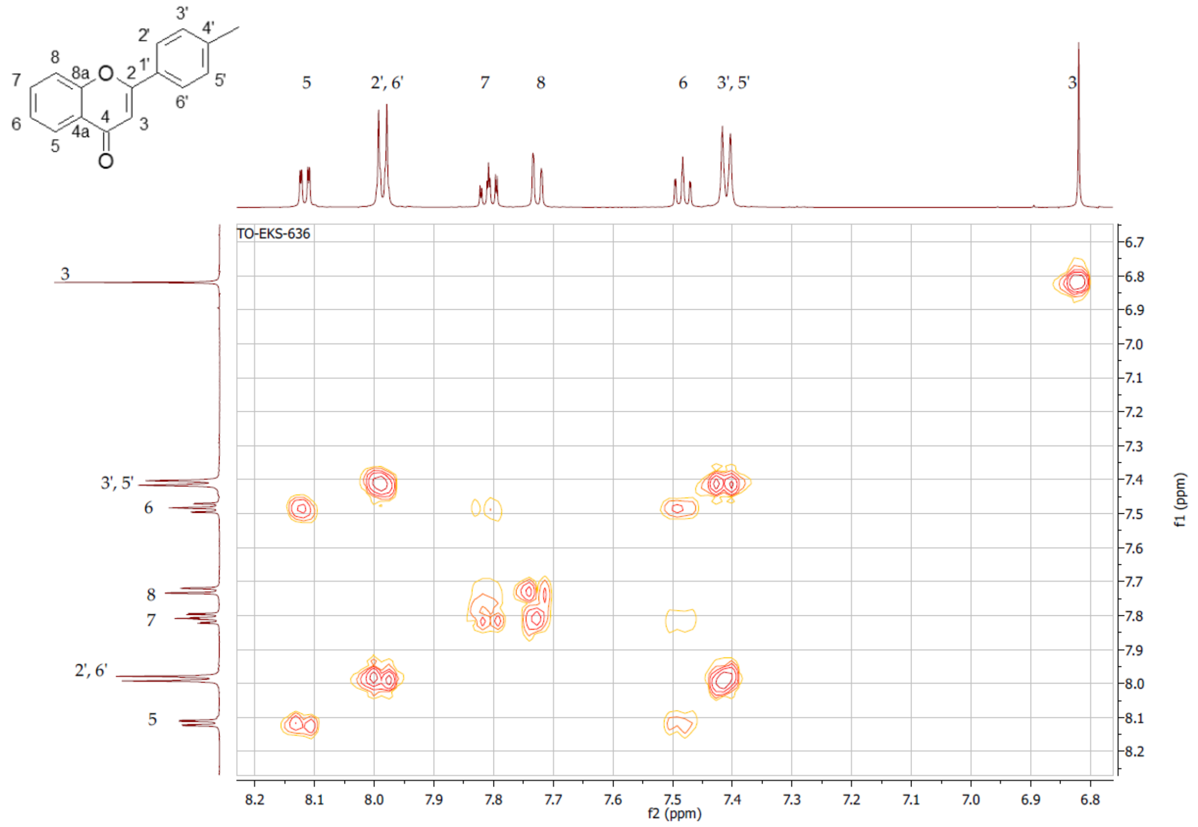

Figure S33. COSY NMR spectrum of 4′-methylflavone (**5**) (Acetone-d6, 600 MHz)

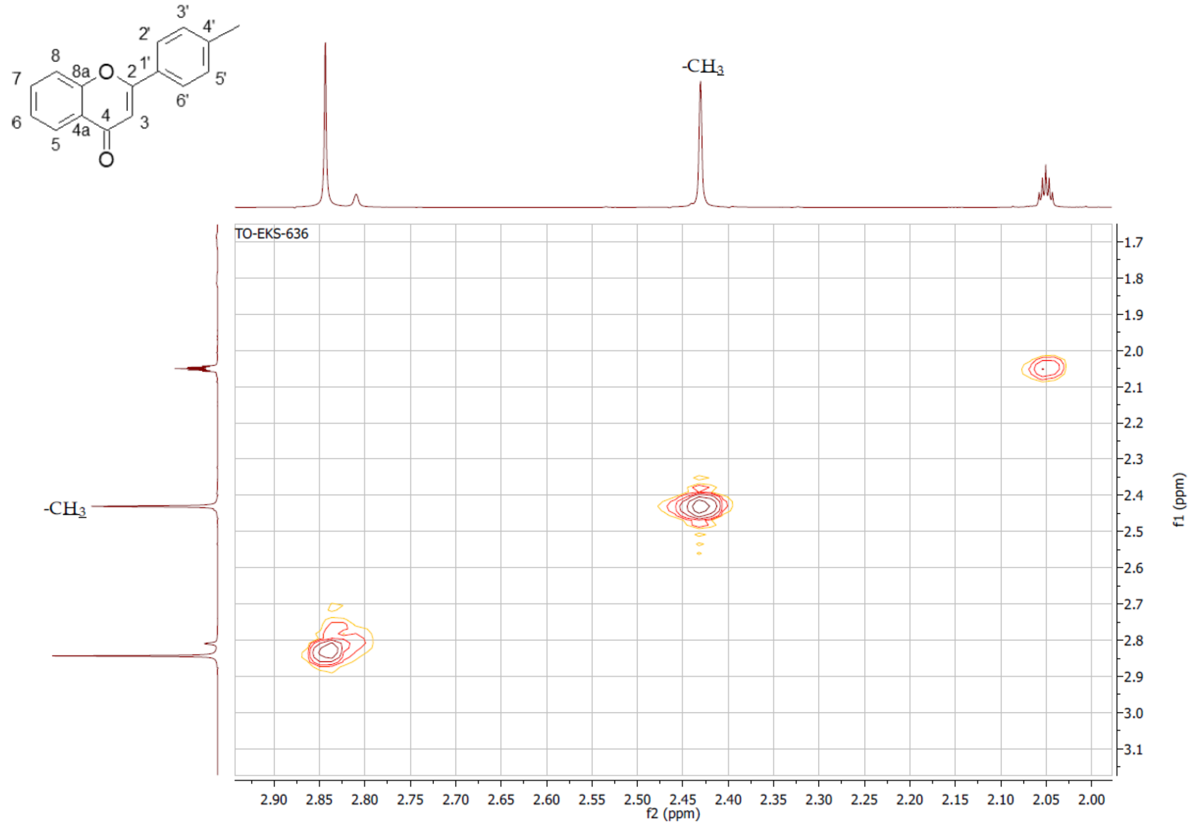

Figure S34. COSY NMR spectrum of 4′-methylflavone (**5**) (Acetone-d6, 600 MHz)

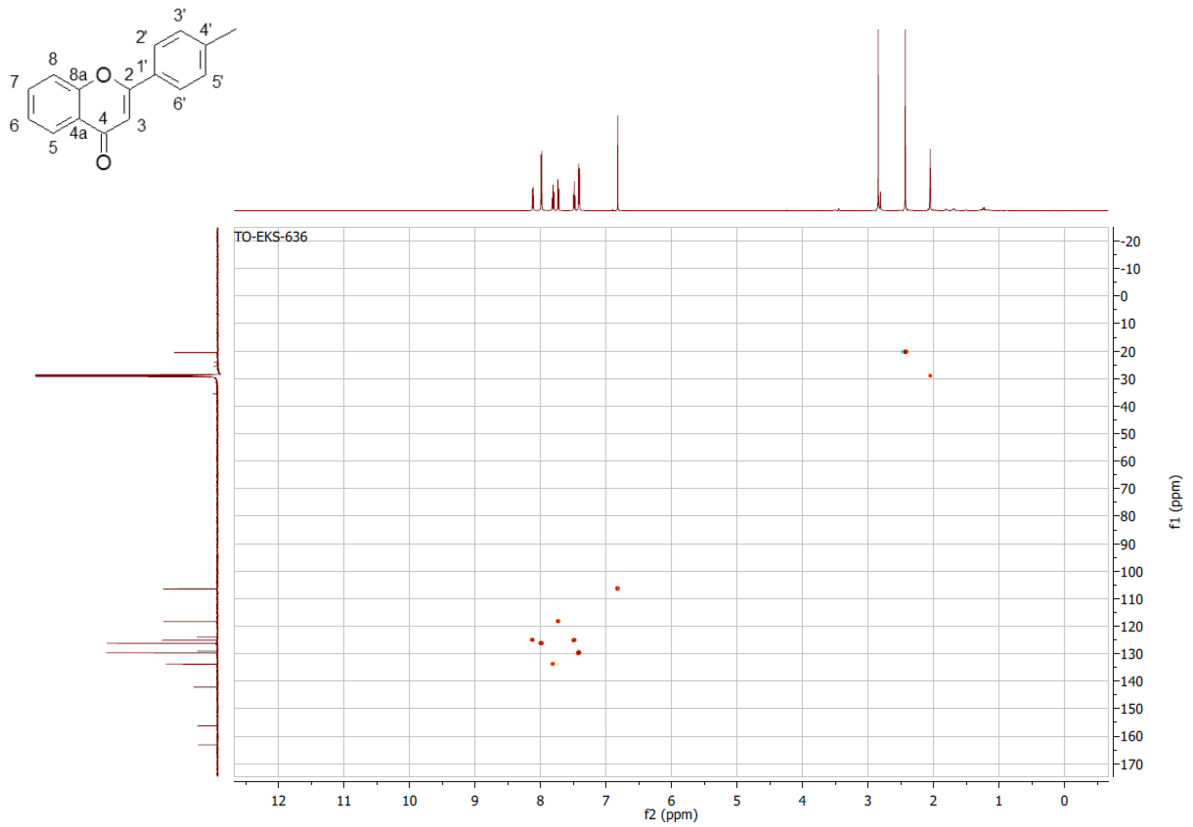

Figure S35. HSQC NMR spectrum of 4′-methylflavone (**5**) (Acetone-d₆, 151 MHz)

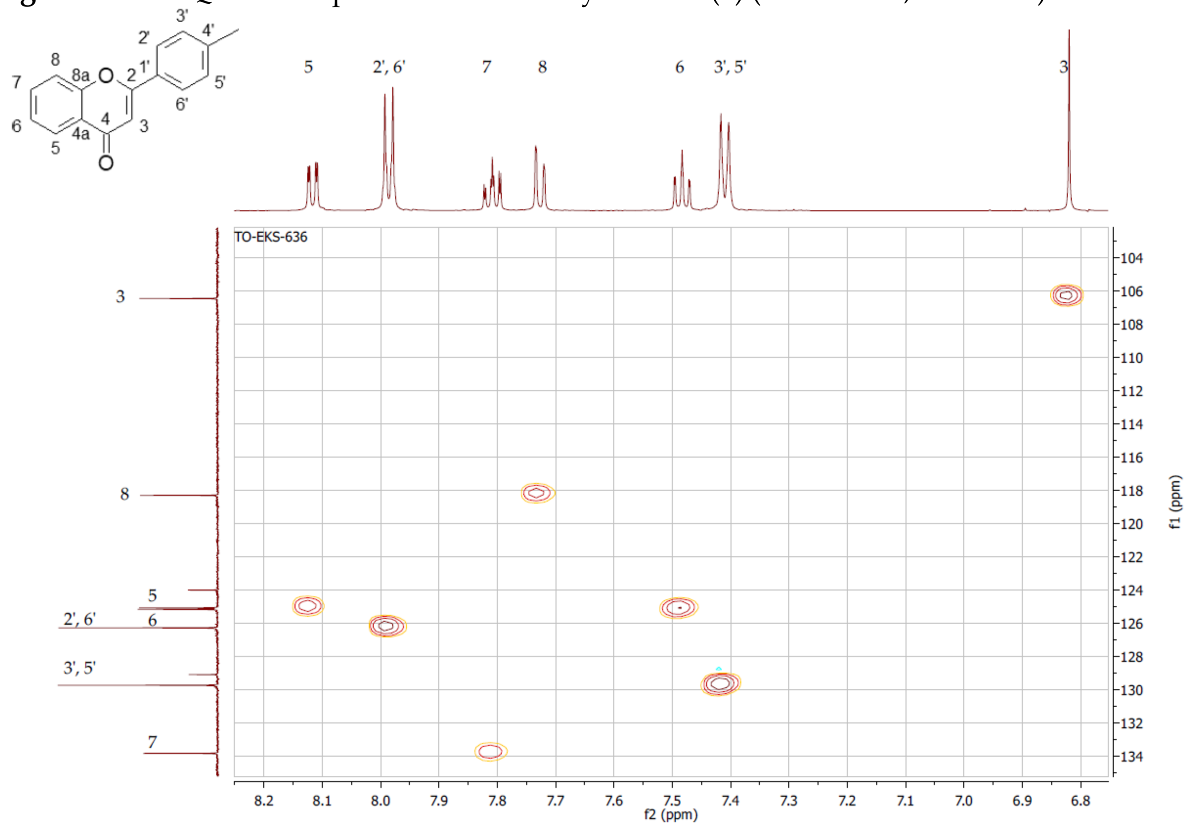

Figure S36. HSQC NMR spectrum of 4′-methylflavone (**5**) (Acetone-d₆, 151 MHz)

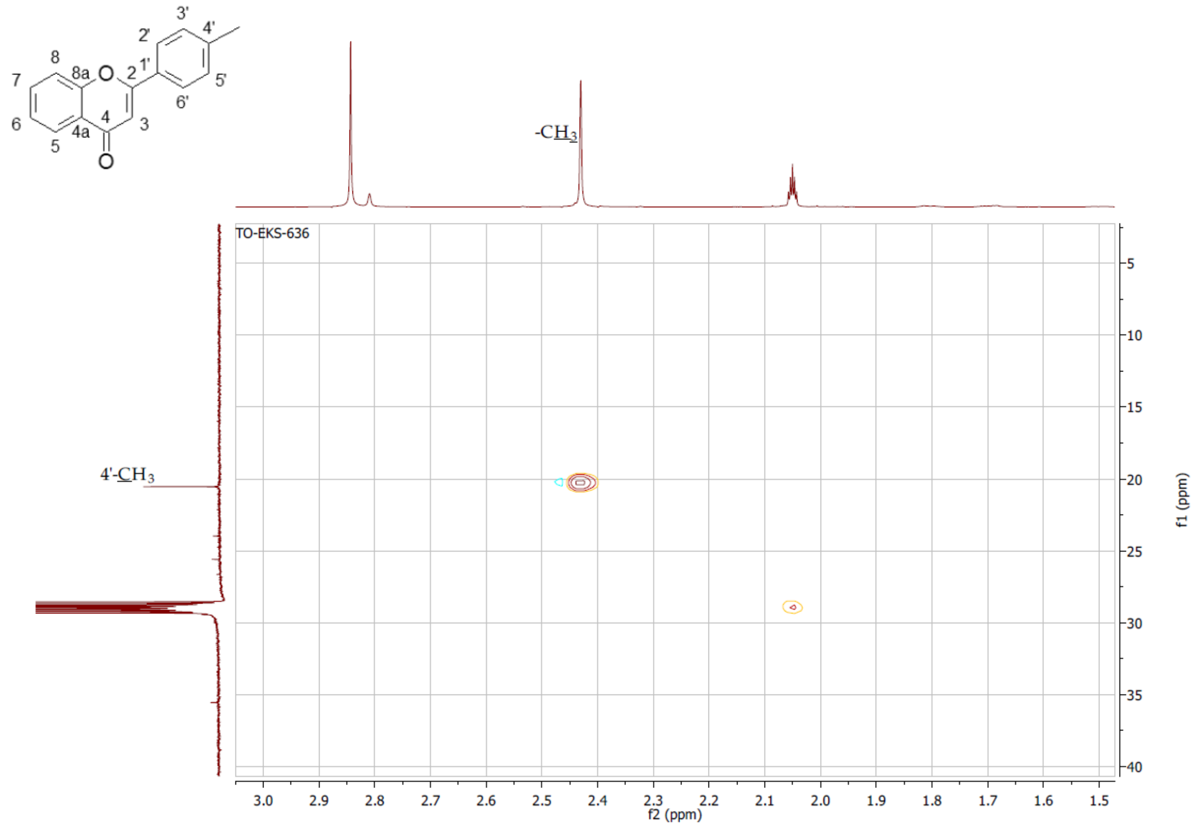

Figure S37. HSQC NMR spectrum of 4′-methylflavone (**5**) (Acetone-d₆, 151 MHz)

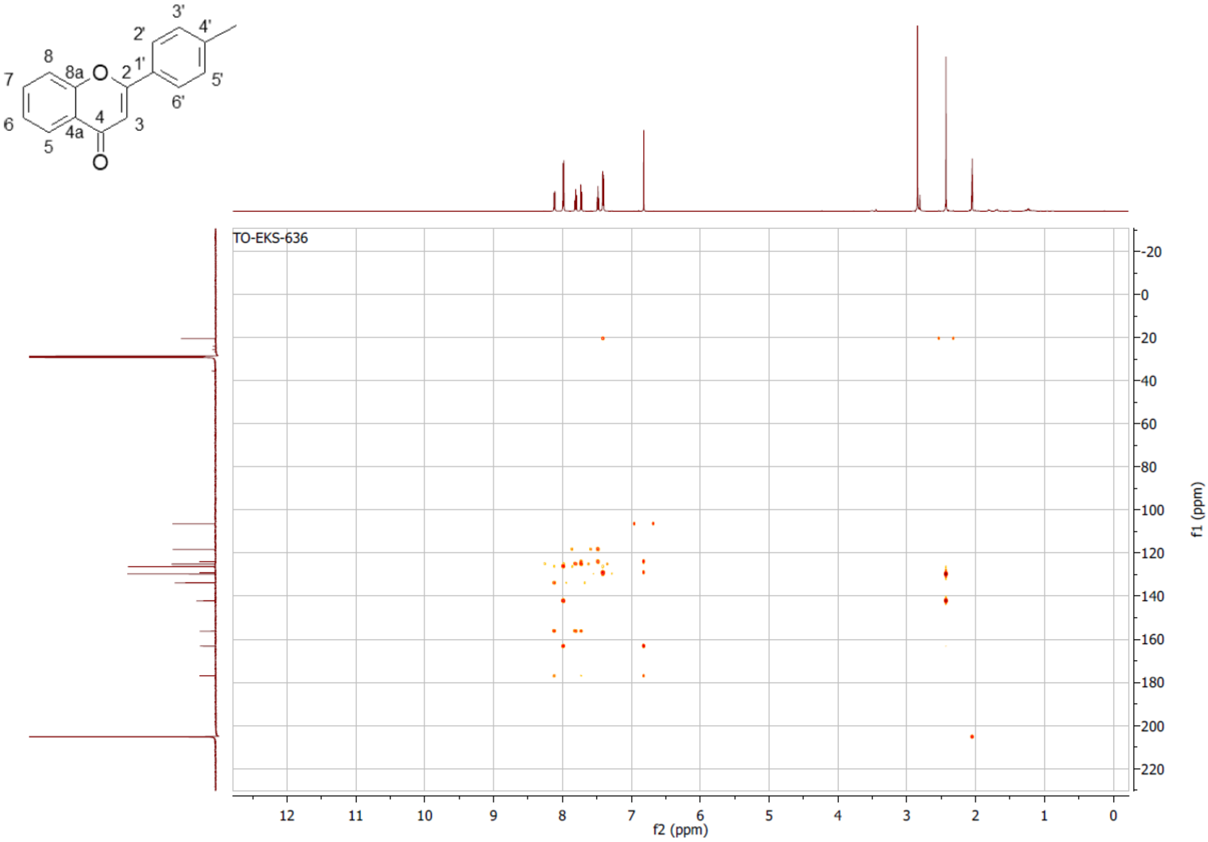

Figure S38. HMBC NMR spectrum of 4′-methylflavone (**5**) (Acetone-d₆, 151 MHz)

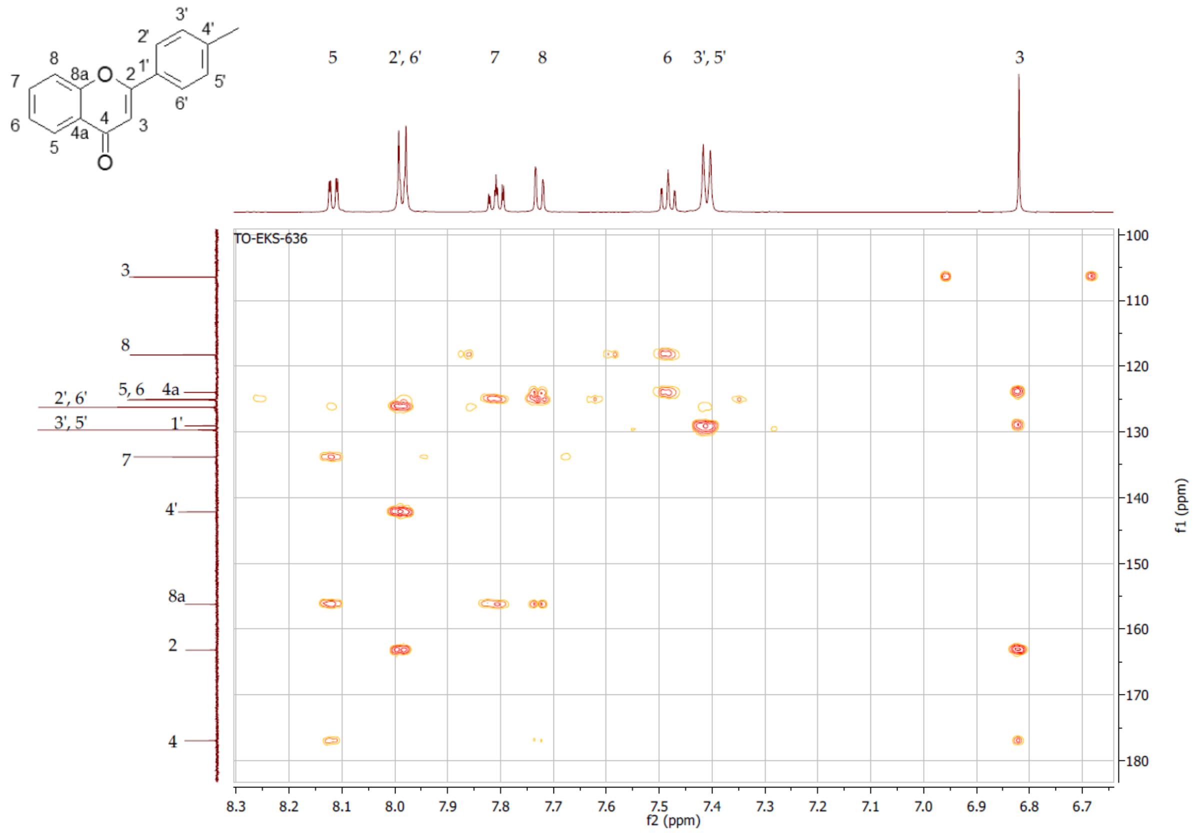

Figure S39. HMBC NMR spectrum of 4′-methylflavanone (**5**) (Acetone-d6, 151 MHz)

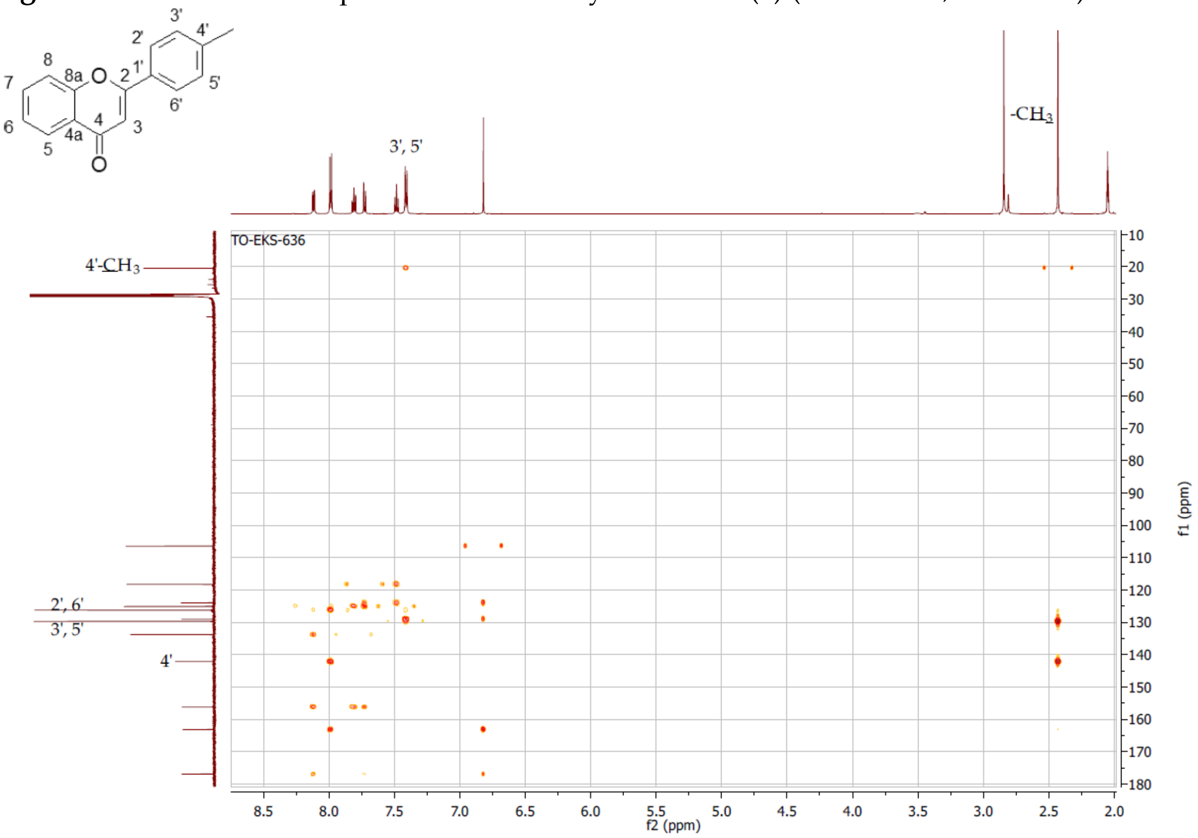

Figure S40. HMBC NMR spectrum of 4′-methylflavanone (**5**) (Acetone-d6, 151 MHz)

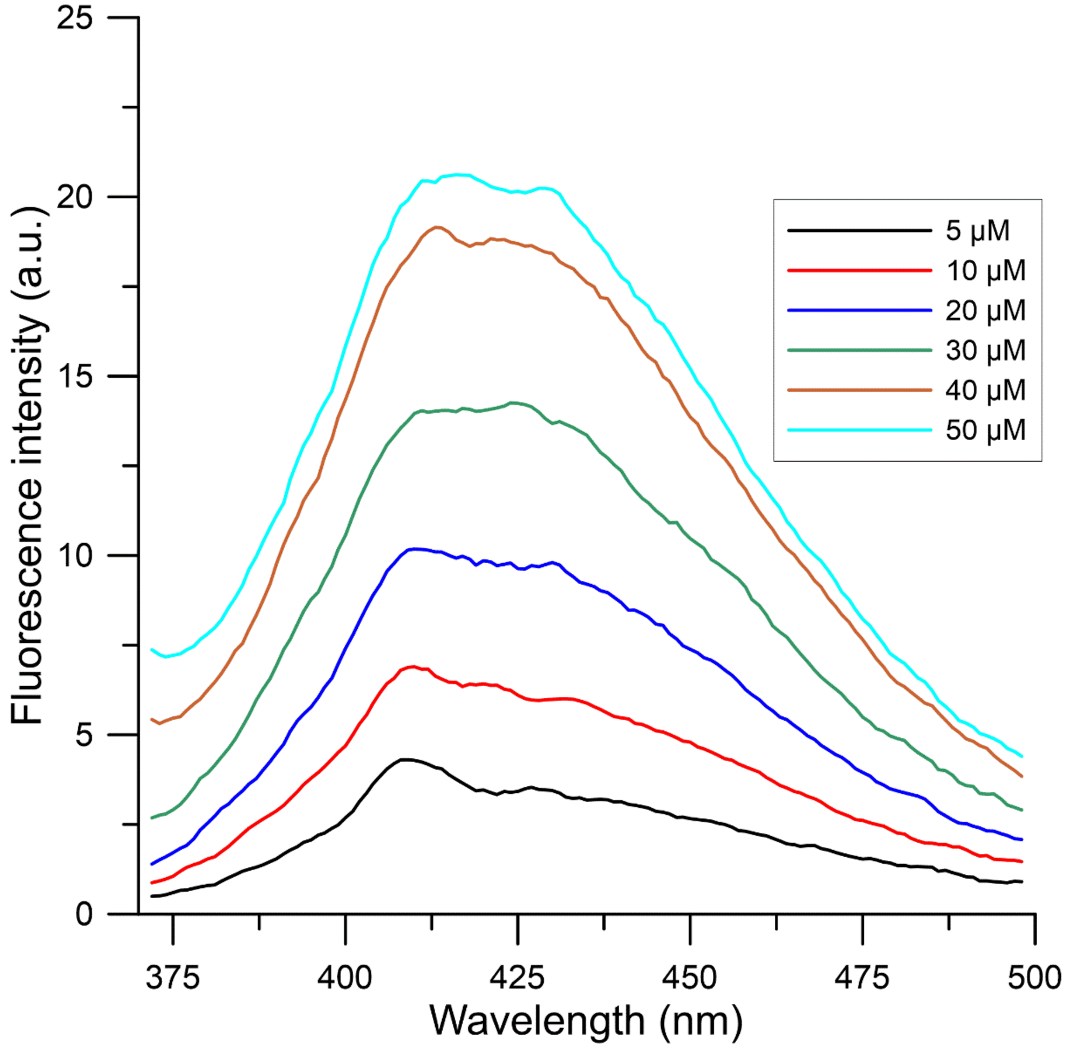

Figure S41. The fluorescence intensity of 4′-methylflavanone, λ_{ex}=360 nm.

Table S1. Selected bands of ATR-FTIR spectra of compounds/RBCMs system and RBCMs, concentration of compounds 25 μM.

	RBCMs	RBCMs+ 2'-hydroxy-4-methylchalcone (**3**)	RBCMs+ 4'-methylflavanone (**4**)	RBCMs+ 4'-methylflavone (5)
	wavenumber (cm^{-1})			
$\nu_{as}(CH_3)$	2955.30±0.14	2955.13±0.10	2955.45±0.09	2954.87±0.30
$\nu_{as}(CH_2)$	2922.82±0.40	2922.17±0.05	2922.31±0.30	2923.63±0.22
$\nu_s(CH_3)$	2869.22±0.22	2869.22±0.02	2869.36±0.18	2868.71±0.40
$\nu_s(CH_2)$	2852.19±0.29	2851.84±0.03	2852.03±0.21	2853.41±0.84
$\nu(C{=}O)$	1739.57±0.10<br>1734.12±0.03	1739.51±0.01<br>1733.91±0.01	1738.26±0.17<br>1732.67±0.87	1739.60±0.01<br>1733.91±0.49
$\nu_{as}(PO_2^-)$	1235.18±0.33	1235.89±0.07	1235.62±0.13	1234.15±0.04
$\nu_s(PO_2^-)$	1064.34±0.04	1064.26±0.05	1064.27±0.08	1064.19±0.10
$\nu_{as}(C\text{-}N^+(CH_3))$	971.47±0.11	971.50±0.10	971.19±0.17	970.59±0.81
$\nu_s(C\text{-}N^+(CH_3))$	925.61±0.01	925.63±0.01	925.58±0.04	925.33±0.24

vibrations: ν - stretching; *as*-asymmetric, *s*-symmetric
